# Single‐Cell Integration of Mouse Models of Autism Reveals Conserved Transcriptomic Signatures of Excitatory‐Inhibitory Imbalance and Glial Dysfunction

**DOI:** 10.1002/glia.70225

**Published:** 2026-10-21

**Authors:** João V. Nani, Victor J. Duque, Alysson R. Muotri, André S. Mecawi

**Affiliations:** ^1^ Department of Biophysics, Escola Paulista de Medicina (EPM) Universidade Federal de São Paulo (UNIFESP) SP Brazil; ^2^ Department of Pediatrics and Department of Molecular and Cellular Medicine University of California, San Diego La Jolla California USA

**Keywords:** computational biology, disease modeling, integrative transcriptomics, intercellular signaling, neurodevelopmental disorders

## Abstract

The etiological heterogeneity of autism spectrum disorder (ASD) complicates identifying core molecular mechanisms. We systematically integrated scRNA‐seq data from 11 diverse genetic and environmental mouse ( *Mus musculus* ) models, encompassing > 300,000 cells across multiple brain regions and developmental stages. Using advanced computational harmonization (RPCA), we identified robust convergent transcriptomic signatures that transcend model‐specific variability. Our findings reveal widespread alterations in neuronal communication pathways, specifically increased excitatory‐inhibitory signaling involving PTN, SLIT, and PTPR pathways. Furthermore, cell–cell communication analysis via NicheNet prioritized ligand‐receptor pairs associated with altered circuit excitability, offering a predictive blueprint of dysregulated intercellular crosstalk. Crucially, we identified significant glial involvement characterized by astrocyte function inhibition, marked by *Aqp4* and *Kcnj10* downregulation related to impaired water/potassium homeostasis and mature oligodendrocyte metabolic dysregulation through *Olig* family upregulation and enhanced oxidative phosphorylation. Differentially expressed genes (DEGs) significantly overlapped with SFARI‐listed high‐confidence risk genes, particularly within excitatory neuronal subtypes. Comparison with human postmortem single‐nucleus data from the prefrontal and parietal cortices demonstrated conserved signatures related to impaired synaptic protein synthesis (GRIN2B, CAMK2A) and oligodendrocyte‐mediated neurodevelopmental deficits (NRXN, DLGAP). This study confirms that E/I imbalance and glial dysfunction are universal cellular pathologies in ASD, providing a robust, large‐scale reference for identifying novel therapeutic targets with single‐cell resolution.

## Graphical abstract

- Integrated scRNA‐seq from 11 ASD models reveals convergent E/I imbalance and glial dysfunction.
- Conserved astrocyte and oligodendrocyte dysregulation transcend model‐specific variability.
- Validated by human datasets.

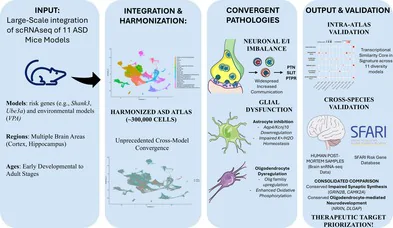

## 1 Introduction

Autism spectrum disorder (ASD) is a complex neurodevelopmental condition defined by a wide range of social, communicative, and behavioral challenges (Hirota and King 2023). It is estimated that about 1 in 100 children around the globe receive an ASD diagnosis (Zeidan et al. 2022), and this prevalence has risen in recent years (Palinkas et al. 2019). Although the exact causes and mechanisms of ASD remain largely unknown, reflecting its multifactorial complexity and variability, different individuals show significant variations in symptom severity and treatment response (Hodges et al. 2020).

Genome‐wide association studies (GWAS) have identified genetic variants associated with ASD, including highly penetrant mutations in genes such as *CHD7*, *SHANK3*, and *FOXG1* (Casanova et al. 2016). However, most cases likely arise from complex interactions among multiple small‐effect genes and environmental factors, including perinatal complications (Karimi et al. 2017). These factors contribute to the disorder's heterogeneity and further complicate efforts to establish a biological basis for ASD. Current ASD treatments, including behavioral therapy and pharmacological interventions that target specific symptoms, fall short of addressing the underlying biological mechanisms. This gap leaves a critical shortfall in our understanding and management of this disorder (Hirota and King 2023) and underscores the importance of innovative approaches to studying ASD.

Recent advances in sequencing technologies, particularly single‐cell RNA sequencing (scRNA‐seq), have unlocked new opportunities for investigating transcriptomic changes (Chehimi et al. 2023) in the brain associated with ASD, where molecular alterations may occur in discrete subsets of neurons or glial cells that are often missed in bulk tissue analyses. Studies indicate that ASD pathogenesis is linked to transcriptomic changes in upper‐layer excitatory neurons that correlate with clinical severity (Velmeshev et al. 2019), reflecting altered synaptic signaling that connects cortical circuits to ASD‐related behaviors (Wamsley et al. 2024) and a widespread dysregulation of these circuits across multiple cortical regions along an anterior–posterior gradient (Gandal et al. 2022).

While those studies using brain samples from individuals with ASD have provided valuable insights, postmortem analyses often reflect later life stages, potentially missing critical changes during neurodevelopment (Fetit et al. 2021). Furthermore, factors that can alter gene expression, such as prolonged medication use (LeClerc and Easley 2015), neurodegenerative processes, and other comorbidities (Kern et al. 2013), can confound the results, making it difficult to isolate changes solely associated with ASD. Thus, animal models, often developed through ASD‐implicated gene manipulations (e.g., knockout/overexpression), have been instrumental in ASD research, offering insights into genetic and environmental components related to specific brain regions (Sierra‐Arregui et al. 2020). However, animal model studies are often limited to single experimental conditions or genetic modifications, and differences in methodologies complicate cross‐study comparisons and data integration (Ryu et al. 2023). Recent bioinformatic advances, particularly algorithms for correcting batch effects while preserving biological signals, present an opportunity to identify conserved differentially expressed genes (DEGs) across various experimental paradigms (Zhou et al. 2023; Hrovatin et al. 2023), brain regions (Gandal et al. 2022), and developmental stages (Kim et al. 2020). Such integrative analyses can uncover shared molecular pathways central to ASD by leveraging diverse experimental designs.

Here, we integrated scRNA‐seq datasets from 11 distinct animal models representing diverse genetic and environmental factors relevant to ASD, encompassing over 300,000 cells across various brain regions and developmental stages. Using advanced computational methods for data harmonization, cell‐type identification, differential gene expression analysis, and inference of cell–cell communication networks, we aimed to identify convergent molecular alterations associated with the ASD physiopathology. Our findings reveal robust, conserved transcriptomic signatures across these models, notably highlighting widespread dysregulation of excitatory‐inhibitory neuronal communication networks and glial dysfunction in astrocytes and mature oligodendrocytes. The clinical relevance of these signatures was underscored by significant overlap with known ASD risk genes from the SFARI database and concordance with transcriptomic alterations in human postmortem brain studies. This integrative study provides a unified framework that transcends model‐specific variability, identifying shared molecular mechanisms and cellular pathologies central to ASD.

## 2 Methods

### 2.1 Data Acquisition and Processing

We curated scRNA‐seq data from 11 independent studies investigating animal models of ASD (Table 1). These studies spanned multiple brain regions, developmental timepoints, and genetic or pharmacological ASD models. Our inclusion criteria encompassed established high‐confidence risk genes (e.g., *Shank3*, *Ube3a*), environmental models (*VPA*), and models of specific neurodevelopmental pathways implicated in ASD pathophysiology. Specifically, we included *Setd1a* models, traditionally linked to schizophrenia (but also ASD), to investigate shared neurodevelopmental deficits and synaptic dysregulation common to both disorders. Similarly, the *Tmem173* (STING) and *Crlf3* models were selected to probe the contributions of neuroinflammatory signaling and cytokine‐related pathways, respectively, which are increasingly recognized as relevant to ASD etiology despite variable behavioral penetrance in mice. This diverse compilation allows for the isolation of core transcriptomic alterations that persist across distinct genetic and environmental insults. All data were obtained from public repositories, and if only raw (fasta/fastq) was available, sequencing data were processed through the 10× Genomics CellRanger platform to perform alignment and form corresponding matrix to perform bioinformatic analysis. For datasets processed in‐house, raw FASTQ files were aligned and quantified using Cell Ranger v7.0 with default parameters mapped to the mm10 reference genome. For studies where count matrices were provided by authors, we verified the compatibility of gene nomenclature with the mm10 reference to ensure consistent merging.

**TABLE 1:** List of studies used to construct the integrated object of animal models for ASD.

References	Brain region	Age	Model	Year	Plataform	Methods
Zhang et al. (2020)	Cerebral cortex	P0	*Sting* Conditional Knockout	2020	10× Genomics	Single‐cell RNA sequencing
Chen et al. (2022)	Prefrontal cortex and striatum	3 months	*Setd1a* haploinsufficient	2022	10× Genomics	Single‐cell RNA sequencing
Donnard et al. (2022)	Cerebral cortex	P5	*Fmr1* Knockout	2022	InDrop	Single‐cell RNA sequencing
Ba et al. (2023)	Hippocampus	Embryo (17.5 days)	*Foxg1* Knockout	2023	10× Genomics	Single‐cell RNA sequencing
Ji et al. (2023)	Prefrontal cortex	3–4 months	*Gls1* Conditional Knockout in *CamKIIα* + neurons	2023	GEXSCOPE	Single‐nucleus RNA sequencing
Zhang et al. (2024)	Hippocampus	3 months	Prenatal exposure to Valproic Acid	2023	10× Genomics	Single‐cell RNA sequencing
Xing et al. (2023)	Cerebral cortex	Embryo (14.5 days) and P0	*Ube3a* gain‐of‐function	2023	Drop‐sec	Single‐cell RNA sequencing
Wilson et al. (2023)	Hippocampus	1 month	*Crlf3* Variant	2023	10× Genomics	Single‐cell RNA sequencing
Ghirardini et al. (2023)	Cerebral cortex	3 months	*Slc6a8* Knockout	2023	10× Genomics	Single‐cell RNA sequencing
Pollina et al. (2023)	Hippocampus	1 month	NPAS4:NuA4 Conditional Knockout	2023	10× Genomics	Single‐nucleus RNA sequencing
Dugger et al. (2023)	Cortex and hippocampus	P0	*Hnrnpu* haploinsufficient	2023	10× Genomics	Single‐nucleus RNA sequencing

For each dataset, quality control was performed to remove low‐quality cells. In our QC pipeline, we computed key metrics including the log‐transformed total RNA counts and the log‐transformed number of detected genes and calculated the percentage of mitochondrial gene expression. We applied a custom MAD (Median Absolute Deviation) outlier function (mad_outlier) to flag cells with extreme values (beyond 5 MADs from the median) for the above metrics (Heumos et al. 2023). While metrics were log‐transformed to calculate MAD scores, the filtering thresholds were applied directly to the cell barcodes. Cells flagged as outliers were removed from further analysis. Additionally, cells were filtered based on mitochondrial gene content: only cells with less than 10% mitochondrial genes were retained in single‐cell datasets, whereas a stricter threshold of 1% was applied for single‐nucleus data to exclude nuclei with cytoplasmic contamination or signs of membrane leakage, consistent with established protocols for snRNA‐seq quality control (Osorio and Cai 2021). The distribution of quality control metrics (nFeature_RNA, nCount_RNA, and mitochondrial percentage) before and after filtering is detailed in Figure S1.

### 2.2 Data Integration, Dimensionality Reduction and Clustering

The raw data were merged and then split by the “paper” identifier to retain sample‐specific information. Each individual dataset was normalized using NormalizeData(), and variable features were identified using FindVariableFeatures() with 3000 features. Data were scaled with ScaleData(), and principal component analysis (PCA) was performed on each dataset with 100 PCs computed. These preprocessing steps were applied to all merged datasets. To harmonize data across the 11 studies, we employed a reciprocal principal component analysis (RPCA) integration strategy as implemented in Seurat (v5.0) due to the high number of cells (> 300,000) (Hao et al. 2024). Unlike standard CCA, RPCA is robust against significant dataset‐specific variation, allowing it to identify shared biological states (anchors) across highly diverse samples without forcing disparate cell types to align. This approach effectively corrected for technical and biological batch effects while preserving the conserved biological signals relevant to ASD pathology. The merged Seurat object was used for integration by invoking the function IntegrateLayers() with the RPCA integration method. The original PCA reduction (computed with 100 PCs) was used as input. We next computed a nearest‐neighbor graph using the integrated RPCA reduction over the first 100 PCs. Clustering was performed at a high resolution (30). This parameter choice was empirically driven to prevent the merging of distinct but rare neuronal subtypes (e.g., specific interneuron classes) that are critical for identifying subtle disease‐specific transcriptomic shifts. While high resolution can lead to over‐clustering, we manually curated and merged clusters at the annotation stage based on canonical marker expression hierarchy (Levels 1 to 3) to ensure biologically meaningful cell identities. For visualization, Uniform Manifold Approximation and Projection (UMAP) was run using the integrated RPCA reduction (using PCs 1–100), which allowed for the detailed exploration of the cellular landscape. This high‐resolution clustering strategy facilitated the identification of subtle, cell‐type–specific transcriptional signatures that may be critical for understanding the underlying biology.

Following the initial quality control based on RNA counts and mitochondrial content, we addressed potential technical artifacts arising from doublets (droplets containing two or more cells). We performed doublet detection and removal using the *scDblFinder* package (v1.23.2) on the integrated dataset. To account for sample‐specific variability, the doublet detection was run independently for each sample (“orig.ident”). This analysis identified 21,291 putative doublets (~6.8% of the total cell count), which were subsequently removed from the dataset. All downstream analyses, including dimensionality reduction, clustering, and differential expression testing, were performed exclusively on the remaining 291,916 singlet cells to ensure statistical robustness and minimize technical bias. The distribution of identified doublets across cell types is detailed in Table S1.

To quantitatively assess the local batch alignment and robustness of our data harmonization across the 11 integrated reference cohorts, we computed the integration Local Inverse Simpson's Index (iLISI) for all high‐resolution cellular annotations (Level 3 subtypes) utilizing the LISImetrics framework within the lisi R package. The iLISI metric evaluates the effective number of datasets represented within a cell's local neighborhood in the integrated latent space, providing an automated benchmark of dataset mixing, detailed in Table S2. Concurrently, to formally evaluate whether the post‐integration coordinate space preserved the discrete biological, chronological, and neuroanatomical properties of the reference lineages without artificial collapsing, we constructed spatial and temporal cell‐count contingency matrices. A Pearson's Chi‐squared (χ^2^) test of independence was executed independently on the raw cell‐count tables for both developmental stages and neuroanatomical brain areas to statistically validate the significance of non‐random biological distribution.

### 2.3 Cell‐Type Identification

After clustering, cells were further analyzed for differential gene expression. Using FindAllMarkers(), positive markers were identified for each cluster (with a minimum log‐fold‐change threshold of 0.25). Dimensionality reduction and visualization (via DimPlot()) were performed to assess the distribution of cells by paper and cluster. Hierarchical clustering was conducted in five successive levels, and cell‐type annotations were assigned by examining the expression profiles of classical gene markers using both literature guidance and data‐driven marker identification.

### 2.4 Differentially Expressed Genes Analysis

We also identified DEGs between ASD and control conditions across all levels of cell classification. Differential expression analysis was performed using the Wilcoxon rank‐sum test. *p*‐values were adjusted for multiple comparisons using the Benjamini‐Hochberg (FDR) method, with a significance threshold of adjusted *p*‐value < 0.05. We applied a minimum log2 fold‐change threshold of 0.25 and required genes to be expressed in at least 10% of cells in either group to be considered for testing. Additionally, we compared these DEGs with the enriched genes identified using the FindAllMarkers function for each cluster to assess whether a given DEG ranks among the most significant genes in that cluster.

To ensure the mathematical robustness of our cell‐level differential expression results and address potential pseudo‐replication biases, we cross‐validated our signatures by comparing the primary Wilcoxon rank‐sum test with a sample‐level pseudobulk framework utilizing DESeq2 aggregation. In addition to computing Spearman's correlation coefficients across Log2 Fold Changes to evaluate effect‐size concordance, we systematically repeated both the functional enrichment (Gene Ontology, GO) and disease‐association analyses under the pseudobulk architecture to evaluate structural convergence. Disease‐associated gene overrepresentation was conducted using a Hypergeometric Enrichment Test against the DisGeNet platform across all cell‐type clusters. To visualize the directional magnitude and statistical significance of these pseudobulk‐validated target genes, we generated log2 fold‐change distribution jitter plots, mapping single‐gene coordinates to their respective log10 *p‐*value pseudobulk profile and explicitly identifying top driving risk targets per population.

### 2.5 Functional Enrichment and Annotation

GO and KEGG Pathway Analysis: For each cluster, both enrichment genes in each cluster and DEGs between ASD and control samples were subjected to enrichment analysis using standard GO databases (biological processes (BP), molecular functions (MF), Cellular Components (CC)) and KEGG pathways. Gene symbols were then converted to Entrez IDs using the bitr() function from the clusterProfiler package with the mouse annotation database (org.Mm.eg.db). To avoid statistical bias toward highly expressed global genes, we defined the background for each enrichment analysis as the specific set of genes expressed (> 0 counts in > 10% of cells) within the cell type being analyzed. We computed the number of cells in which each gene was expressed (by summing the counts of cells with nonzero expression) and retained only genes with nonzero expression. This background set was similarly annotated with Entrez IDs. For each cluster, GO enrichment analyses were performed separately for the three ontologies: BP, MF, and CC. The enrichGO() function was used with the following parameters: the Benjamini–Hochberg (BH) method for *p*‐value adjustment, a *p*‐value cutoff of 0.01, and a q‐value cutoff of 0.05. Results were rendered “readable” by converting Entrez IDs back to gene symbols. In parallel, KEGG pathway analysis was conducted using the enrichKEGG() function (with organism set to “mmu” and a *p*‐value cutoff of 0.05) to identify significantly enriched pathways. Enriched terms were visualized using volcano plots and bar charts. IUPHAR and Transcription Factor Classification: DEGs were further categorized based on their classification as transcription factors (TFs) (Hu et al. 2019) or according to physiological and pharmacological criteria using the IUPHAR/BPS Guide to Pharmacology (Armstrong et al. 2020).

### 2.6 Comparison Between Datasets

To assess the consistency of transcriptomic signatures across the studies included in our integration, we performed several comparative analyses. First, we evaluated the concordance between DEGs identified in the integrated object versus those found within subsets representing each individual reference study. This involved correlation analyses at the second level of cell classification, quantifying agreement using Spearman's correlation coefficients and statistical significance. Second, we conducted a shared gene analysis among these reference subsets, examining both the number of overlapping DEGs and the consistency of their regulatory direction (upregulation or downregulation) across studies. Third, to explore cross‐model effects, we assessed the expression patterns of key genes associated with the specific genetic perturbations used in the source studies (*Crlf3, Fmr1, Foxg1, Gls1, Hnrnpu, Npas4, Setd1a, Slc6a8, Tmem173*, and *Ube3a*). We specifically examined the regulation of these genes in each subset and within the valproic acid (VPA) pharmacological model subset derived from our integrated object, evaluating whether this model significantly recapitulated genetic model‐specific alterations at the second level of cell classification. Finally, for a direct comparison with results from an original publication, we selected Donnard et al. (2022), chosen for the compatibility of its reported cell annotations with our integrated dataset. For this specific comparison, neuronal clusters were matched based on our second level of hierarchical classification, while non‐neuronal clusters were compared using the more granular third level. DEGs reported in the original paper were filtered (average log2 fold change > 0.25, adjusted *p*‐value < 0.05), and Venn diagrams were generated to visualize the overlap and directional consistency with DEGs identified in our integrated object for corresponding cell types.

### 2.7 Cell–Cell Communication Analysis

We used CellChat (Jin et al. 2021) to infer and compare cell–cell communication networks across the integrated dataset. We utilized the CellChatDB mouse database for ligand‐receptor interactions. Significant interactions were identified using a permutation test (*n* = 100), with a *p*‐value threshold of < 0.05. The probability of communication was calculated using the law of mass action based on the average expression values of a ligand by a sender cell group and a receptor by a receiver cell group.

To ensure that the comparative weights and inferred interaction strengths reflect true transcriptomic signaling dynamics rather than numerical confounding factors, such as absolute cell numbers, fluctuating subtype proportions, or dataset composition imbalances, the communication probability was computed by executing the computeCommunProb function with the population.size = FALSE parameter. This internal algorithmic control isolates ligand‐receptor expression magnitude from cell‐type abundance, rendering the calculated interaction values mathematically invariant to cluster size or density variations across experimental conditions.

The analysis was performed in two stages: Global Analysis: All cell types were considered to construct an overall communication network, where nodes represent cell types and edge thickness indicates the strength of signaling interactions, at the second level of the hierarchical clustering. Condition‐Specific Analysis: Separate networks were constructed for ASD and control conditions, both in all clusters in the second level and only in the excitatory and inhibitory neurons in the third level on the hierarchical clustering. Specific signaling pathways (e.g., VEGF, PTN, SLIT, PTPR and SEMA3) were further examined.

### 2.8 Ligand‐Receptor Interaction and NicheNet Analysis

To dissect the molecular underpinnings of altered cell–cell communication between inhibitory (sender) and excitatory (receiver) neurons, we employed NicheNet (Browaeys et al. 2020). This approach predicted the ligand–receptor interactions and downstream target genes that mediate the observed transcriptional changes. Ligand Prioritization: Ligands were ranked based on the area under the precision‐recall curve (AUPR) and their fold change between ASD and control conditions. Receptor Analysis: A complementary heatmap was generated to display the expression and predicted interaction strength of key receptors in excitatory neurons. Target Gene Prediction: Predicted target genes in excitatory neurons, as influenced by top inhibitory neuron ligands, were visualized using heatmaps that indicate regulatory potential.

### 2.9 Validation and Characterization of DEGs With External Databases

To further validate and characterize the DEGs identified between ASD and control conditions (primarily focusing on the third level of hierarchical cell classification), we first assessed their clinical relevance by cross‐referencing them with the Simons Foundation Autism Research Initiative (SFARI) Gene database (Abrahams et al. 2013). For each DEG present in the SFARI database, we extracted its associated evidence score and source, comparing these against the gene's average log2 fold change (avg_log2FC) within each relevant cell cluster. Functional enrichment analysis, including GO and KEGG pathways and classification as TFs or according to physiological/pharmacological criteria using the IUPHAR/BPS was subsequently performed specifically on this subset of SFARI‐matched DEGs, following the procedures previously described. Additionally, to investigate patterns of commonality versus cell‐type specificity in gene dysregulation, we analyzed the distribution of all DEGs across the third‐level clusters. We identified the top 20 most frequently shared DEGs across clusters and the top 10 DEGs uniquely regulated within a single cluster, visualizing their regulation status.

To evaluate the translational relevance of the transcriptomic alterations identified in our integrated animal model dataset, we compared DEG profiles with those reported in a large human postmortem single‐nucleus RNA‐seq study of ASD by Gandal et al. (2022). We obtained the published DEG lists from the Gandal et al. study for major cell types across the prefrontal cortex, parietal cortex, and occipital cortex, including gene identity, average log2 fold change, and statistical significance. Cell type annotations were manually matched between our integrated dataset and the Gandal et al. study to ensure comparability across major neuronal and non‐neuronal populations. For each matched cell type within each cortical region, we identified the set of common DEGs (shared DEGs) by finding the intersection between the significant DEG lists from our integrated dataset and the corresponding list reported by Gandal et al. (2022). We then manually calculated the number and percentage of these shared DEGs that exhibited concordant regulation (same direction of avg_log2FC change in both datasets). Furthermore, we computed Spearman's correlation coefficient between the avg_log2FC values of the shared DEGs obtained from our integrated dataset and those reported by Gandal et al., determining the statistical significance (p‐value) of this correlation. These comparisons were visualized for each cortical region using Venn diagrams to depict DEG counts and overlap, alongside scatter plots illustrating the correlation of avg_log2FC values for shared DEGs, with statistical significance. For shared DEGs in the parietal cortex, we conducted cell‐type‐specific functional enrichment using SynGO (Koopmans et al. 2019) for synaptic genes in excitatory and inhibitory neurons, and Metascape (Zhou et al. 2019) for mature oligodendrocytes.

To formally test whether the intersections observed between our cell‐type‐specific DEGs and external reference datasets were significantly greater than expected by chance, we performed an overrepresentation analysis via a formal Hypergeometric Enrichment Test. The statistical model evaluated the probability of drawing the observed number of overlapping genes from the specific DEG pool given the background gene universe. For the cross‐species translational assessment against human cortical datasets (Gandal et al. 2022), the hypergeometric test evaluated strictly directionally concordant genes (co‐upregulated or co‐downregulated targets across both species; Up/Up or Down/Down) to ensure rigorous biological alignment. Multiple‐testing correction was programmatically applied across all tested lineages using the Benjamini‐Hochberg False Discovery Rate (FDR) method, with a strict significance threshold of FDR < 0.05.

### 2.10 Statistical Analyses

For all differential expression and enrichment analyses, statistical significance was determined using adjusted *p*‐values (e.g., Benjamini–Hochberg correction). Spearman's correlation coefficients were calculated to evaluate the concordance between DEG sets across datasets and conditions. Graphical representations (e.g., UMAPs, volcano plots, dot plots, heatmaps, and Venn diagrams) were generated in R using ggplot2 and related packages or using GraphPad Prism 8 (GraphPad Software, San Diego, CA, USA).

## 3 Results

### 3.1 Characterization of the Integrated Dataset of Animal Models for ASD

We successfully integrated data from 11 studies using advanced methods in Seurat (v5.0). The integrated dataset comprises 155.132 cells from ASD models and 158.075 cells from control animals (Figure 1A). Hierarchical clustering classified cell types across functional and transcriptomic levels were accurately performed (Table S3, Figure S2). Figure 1B shows cell distribution across metadata categories, demonstrating a generally uniform distribution. The temporal distribution of cell subtypes confirmed excellent preservation of maturation profiles; for example, immature progenitor populations remained strictly tied to early stages, with Granule Neuroblasts comprising 71.66% Embryo and 26.60% Early Postnatal cells (0.79% Adult), and Neuronal Intermediate Progenitors mapping to 52.86% Embryo and 39.01% Early Postnatal fractions. Spatial contingency mapping further validated neuroanatomical fidelity post‐integration, with cortical intratelencephalic projection clusters retaining their target layers, with L6 IT neurons mapping to 88.42% Cortex and 8.27% Hippocampus (0.05% in Striatum), and L4/5 IT cells showing heavy enrichment in the Cortex (48.75%) and Prefrontal Cortex (27.18%). The Pearson's Chi‐squared test of independence on the raw cell‐count contingency matrices confirmed a highly significant, non‐random biological sorting across the integrated space for both developmental stages (χ^2^ = 259,263.3, df = 63, *p* < 2.2 × 10^−16^) and neuroanatomical brain areas (χ^2^ = 158,167.4, df = 84, *p* < 2.2 × 10^−16^). Taken together, these metrics mathematically support that our RPCA pipeline decoupled technical batch artifacts while preserving the native spatial–temporal landmarks of the mammalian brain (Figure S3A,B and Table S4). To ensure transparency regarding dataset contribution, the distribution of cells across each model, condition (ASD vs. Control), and cell type is also detailed in Table S5. While some variability in cell numbers exists, the RPCA integration ensured that all models contributed to the defined clusters.

**FIGURE 1:**
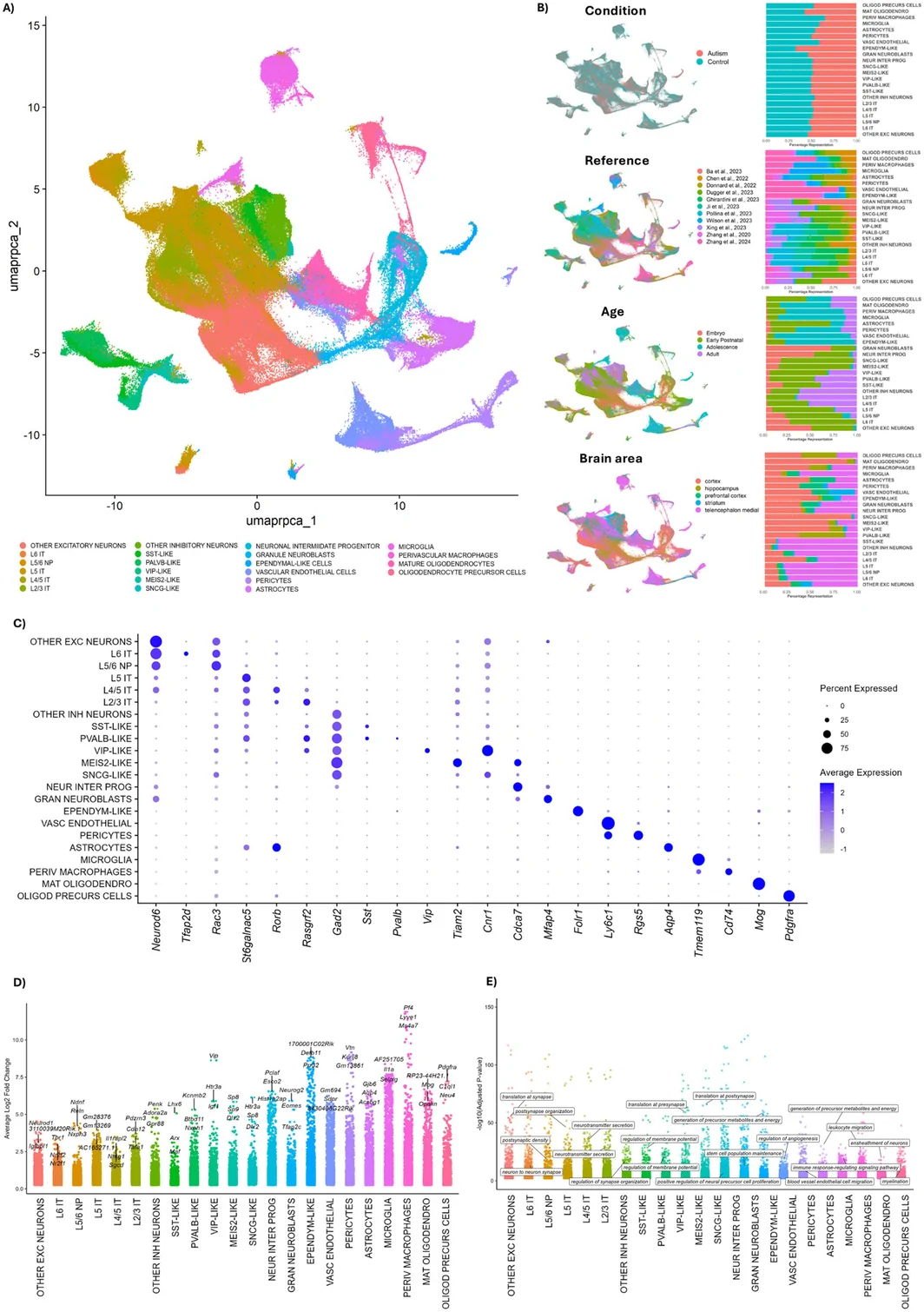
Integration and Analysis of Single‐Cell RNA sequencing from mice models of ASD. (A) UMAP plot displaying clusters after reciprocal PCA (RPCA) integration of single‐cell RNA sequencing data from 11 different studies. The plot shows the third level of hierarchical clustering, highlighting distinct cell populations identified across the datasets, colored by major cell types. (B) Left: UMAPs demonstrate the distribution of cells grouped by metadata categories: Condition (ASD vs. control), reference (source of the 11 references used in the integration), age (age of the animals used in the experiments), and brain area (regions of the brain where samples were collected). Right: Corresponding bar plots depict the percentage representation of each feature within each cell identification cluster from Panel A. (C) Dot plot illustrates the expression levels of key gene markers used to classify each cluster identified at the third level of hierarchical clustering. The dot size represents the proportion of cells expressing the gene, and the color intensity indicates the expression level. (D) Distribution plot illustrates the top three most differentially expressed genes (average log2 fold change) in each cluster at the third level of hierarchical clustering. (E) Distribution plot illustrates all significant gene ontology terms and KEGG pathways, for each cluster at the third level of hierarchical clustering. Relevant terms for each cell type function are highlighted in the figure.

To quantitatively evaluate the robustness of our data harmonization and eliminate the risk of dataset‐driven overcorrection or residual technical batch effects, we computed the Local Inverse Simpson's Index (iLISI) across all cell populations (Figure S3C and Table S2). Our analysis revealed consistent and widespread dataset mixing across virtually all refined cellular subtypes. Key neuronal and non‐neuronal lineages displayed robust batch homogenization, highlighted by high mean iLISI scores in VIP‐like cells (mean iLISI = 3.69) and oligodendrocyte precursor cells (OPCs; mean iLISI = 3.48). Expected neuroanatomical variations were captured in mature cortical projection layers (L2/3, L4/5, and L6 IT), which stabilized within a healthy range of 2.2–2.3, faithfully reflecting the regional specificity of the primary brain dissections. Remarkably, microglia yielded the lowest mixing profile (mean iLISI = 1.78); this low score could reflect profound transcriptomic heterogeneity associated with active versus homeostatic immune states captured across multi‐centric experimental contexts, rather than unresolved computational batch architecture.

Figure 1C displays expression patterns of canonical marker genes for cell type annotation at the third hierarchical level, showing high cell‐type specificity; for instance, *Aqp4* for astrocytes, *Vip* for VIP‐expressing interneurons, and *Gad2* as a pan‐inhibitory marker (Table S6). A distribution plot (Figure 1D) highlights genes positively enriched in various cell types by average log2 fold change, with the top three emphasized (Table S7). GO and KEGG analysis validated cell type classifications (Figure 1E), showing relevant pathways like immune response‐regulating signaling (GO:0002764) in microglia and regulation of angiogenesis (GO:0045765) in vascular endothelial cells (Table S8).

### 3.2 Cell Type Transcriptomic Alterations on Integrated ASD Mice Models

Figure 2A displays DEGs comparing ASD vs. control for each cell type, highlighting *Ttr* and the non‐coding RNA *BC023719*, as highly up‐ and downregulated genes, respectively, in several cell types (Table S9). Notably, *Ttr* encodes transthyretin, a carrier protein responsible for transporting thyroxine (T4) and retinol‐binding protein across the blood–brain barrier. The pyramid chart in Figure 2B quantifies DEGs per cell type, showing total upregulated or downregulated genes and the overall DEG count. Ependymal cells had the largest number of DEGs (7559, mostly downregulated). This chart also indicates the number of DEGs overlapping with positively enriched gene markers on each cell type (Table S10), revealing distinct patterns: in Other Inhibitory Neurons and Astrocytes, 75% and 98% of these overlapping genes were downregulated, respectively. Conversely, in Oligodendrocyte Precursors, 88% were upregulated. Significant correlations between DEGs and enriched genes were observed for several cell types (Table S10). For example, L4/5 IT neurons showed a strong positive correlation (*r* = 0.51, *p* < 0.001; Figure 2C), with *Slc6a7* (which encodes a proline transporter localized in presynaptic plasma membranes) showing the highest positive correlation and *Camk2n1* (an endogenous inhibitor of calcium/calmodulin‐dependent protein kinase II) the most negative. Astrocytes exemplified a significant negative correlation (*r* = −0.17, *p* < 0.001), with the enriched *Aqp4*, a key marker gene encoding a water channel protein primarily anchored at astrocytic end, significantly downregulated in ASD samples (Table S10). DEGs were categorized using IUPHAR/BPS Guide annotations and as TFs (Figure 2D, Table S11). Examples include downregulation of *Comt* (an enzyme responsible for the O‐methylation and degradation of catecholamine neurotransmitters) in Neuronal Intermediate Progenitors and upregulation of *Gad1* (the enzyme that catalyzes the production of gamma‐aminobutyric acid (GABA) from L‐glutamic acid) in SNCG‐like interneurons. Among TFs, *Ebf1*, a transcription factor that regulates neurogenesis and cellular differentiation, was upregulated in Other Excitatory Neurons, L5 Neurons, and Granule Neuroblasts. Widespread alterations in Solute Carrier (SLC) family transporters, membrane proteins responsible for the transmembrane movement of solutes, amino acids, and ions (e.g., *Slc7a5*, *Slc7a14*) were seen in neuronal and non‐neuronal populations. GO and KEGG pathway enrichment analyses on DEGs (Figure 2E, Table S12) revealed enriched brain function GO terms across multiple cell types, with “Cognition” (GO:0050890) emerging in various excitatory/inhibitory neurons and glial cells, and “learning” (GO:0007612) in excitatory neuron subtypes.

**FIGURE 2:**
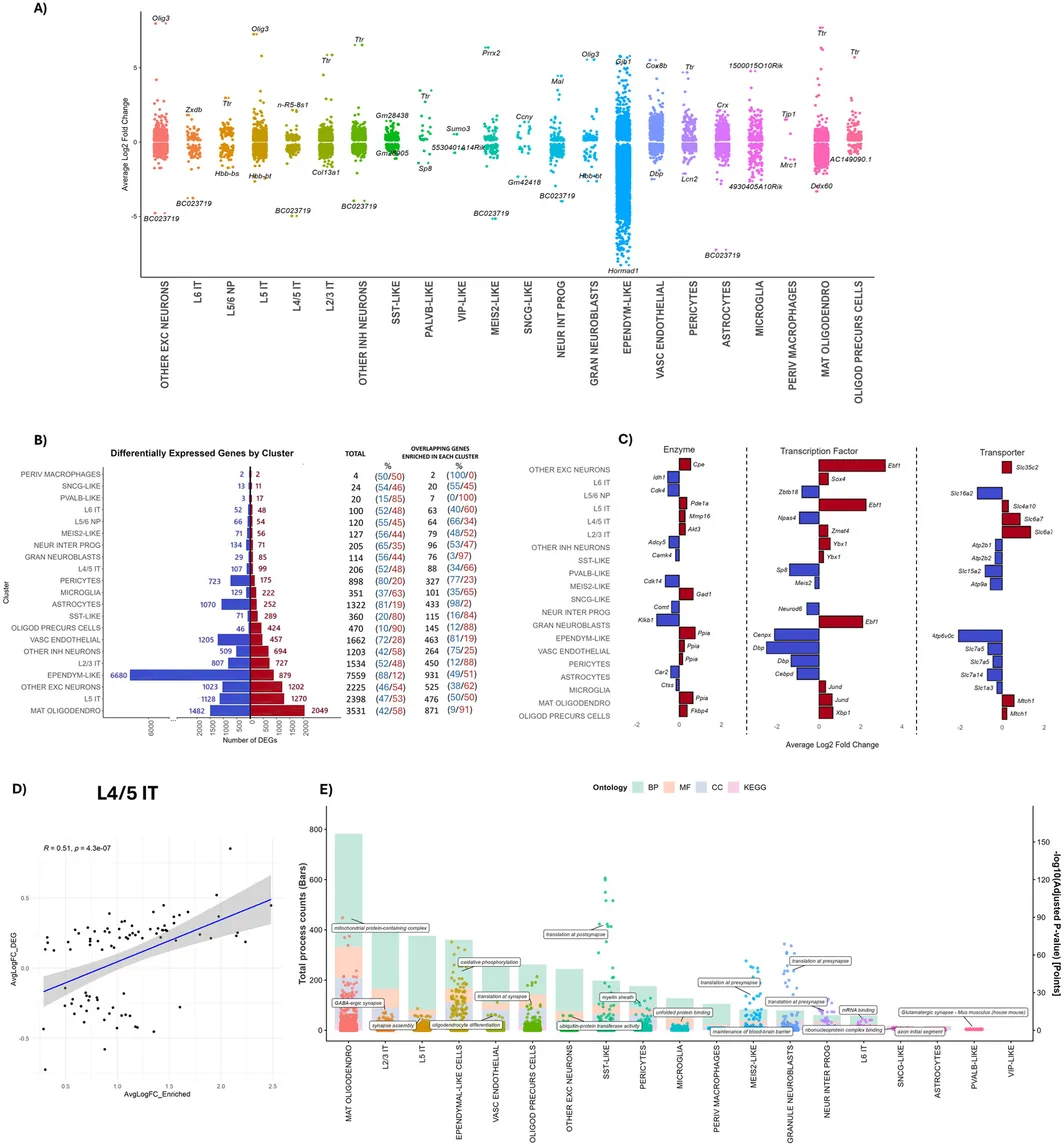
Comparative analysis between ASD and controls in the integrated object third level. (A) The distribution plot illustrates the most upregulated and downregulated genes (by average log2 fold change) in the comparison between samples from ASD and controls for each cell type. (B) The pyramid bar plot shows the burden by clusters with the number of DEGs, ordered by the count of upregulated genes (red) and downregulated genes (blue). The total number of genes is also represented by the percentage of upregulated and downregulated genes, along with the overlap of the enriched genes in each cluster as shown in Figure 1C, representing the percentage of upregulated and downregulated genes again. (C) DEGs are represented according to their identity as transcription factors (Hu et al. 2019), enzymes, or transporters based on their physiological or pharmacological classifications using the International Union of Basic and Clinical Pharmacology and British Pharmacological Society (IUPHAR‐BPS) (Armstrong et al. 2020). (D) An example of Spearman's correlation analysis between DEGs and enriched genes in the cluster L4/5 IT neurons. (E) The GO and KEGG analyses by cluster are represented, showing the total counts (left Y) for each process in the bar plot and each term in the volcano plot, highlighting the most significant term (−log10 adjusted *p*‐value, right Y).

To ensure the robustness of our differential expression results and address potential biases arising from single‐cell statistical methods (pseudoreplication), we performed a validation analysis comparing our primary method (Wilcoxon rank‐sum test at the single‐cell level) with a pseudobulk approach (DESeq2 aggregation at the sample level). We evaluated the concordance of effect sizes specifically for the genes identified as differentially expressed by the single‐cell analysis, and the comprehensive results, including the full pseudobulk output, the significant single‐cell DEGs, and the specific overlap of significant genes between both approaches, are provided in Table S13. We observed a significant positive correlation between the Log2 Fold Changes obtained from both methods across the majority of cell types (Figure S4A), particularly in key populations such as L2/3 IT neurons (*R* = 0.74) and L6 IT neurons (*R* = 0.79). While an expected technical divergence in rank‐metrics within specific populations such as mature oligodendrocytes (*R* = 0.18) and certain interneuron subsets such as PVALB (*R* = 0.3), our conclusions remain robustly supported through high‐level functional convergence. This differential sensitivity is a well‐documented trade‐off in single‐cell genomics, where bulk‐like aggregation tools account for inter‐individual biological variance but suffer from decreased statistical power compared to cell‐level tests.

We also performed independent disease‐association and functional enrichment analyses strictly under the pseudobulk framework. Remarkably, a hypergeometric disease‐overlap analysis against the DisGeNet platform for all clusters (Figure S4B, Table S14) revealed that the systemic engagement of the mature oligodendrocyte lineage in neurodevelopmental pathology is highly reproducible, yielding the most prominent disease‐linked profile among all integrated cell types (FDR = 6.48 × 10^−50^), driven by 55 core disease‐linked genes including *Apoe*, *Cacna1a, Ncor2*, and *Vasp*. Furthermore, our independent pseudobulk functional enrichment analysis successfully identified 161 BP for the oligodendrocyte lineage. Among exact term intersections (Table S15), the core mitochondrial and metabolic signature “Response to Oxidative Stress” (GO:0006979) achieved independent statistical significance in both cell‐level (*p*adj = 2.97 × 10^−4^) and sample‐level pseudobulk (*p*adj = 0.042) runs, alongside robust alterations in pathways tied to “cognition” (GO:0050890) and “learning or memory” (GO:0007611). This indicates that while the pseudobulk approach is less sensitive due to reduced statistical power, the directionality and magnitude of the transcriptional alterations identified by the single‐cell analysis are biologically consistent. Consequently, we proceeded with the Wilcoxon‐derived signatures for downstream functional characterization to leverage the full resolution of the single‐cell data.

### 3.3 Comparison Between Integrated ASD Mouse Models and Individual Datasets

Correlation analysis showed varying concordance between DEGs in the integrated dataset and individual references at the second cell type classification level (Figure S5A, Table S16). Strong positive correlations for inhibitory neurons, excitatory neurons, and progenitor cells across multiple references suggest a conserved transcriptomic dysregulation. To eliminate total DEG count biases across cohorts, a normalized pairwise directional concordance analysis was executed across all seven major cell classes, programming individual matrices based on dataset representation density (Figure S5B). Major neuronal classes (inhibitory and excitatory neurons) along with astro‐ependymal cells displayed high conservation, with multiple pairwise references yielding a strict directional consistency rate between 75% and 100%. Similarly, despite variations in absolute baseline gene counts and limited direct overlap among specific age‐restricted cohorts, whenever pairwise dataset overlaps were present within the precursors and mature oligodendrocytes fraction, they exhibited a consistent directional trend, with shared DEGs predominantly spanning the consistency range across available reference comparisons. Conversely, immune cell and progenitor matrices showed lower overall overlap density, reflecting cohort‐specific sample sizes and chronological age differences across the primary studies.

In the VPA model subset (Zhang et al. 2024), key ASD‐associated genes manipulated in ASD models were consistently downregulated in several cell types (Figure S5C). While *Fmr1* and *Foxg1* exhibited a widespread decrease spanning virtually all cell classes, *Tmem173* (STING) downregulation was restricted exclusively to the immune cell fraction, matching the tissue boundaries reported in its original reference paradigm (Zhang et al. 2020).

To formally validate these transcriptional signatures against an independent statistical architecture, we compared our cell‐type‐specific DEGs with those published by Donnard et al. (2022) using a formal Hypergeometric Enrichment Test on the directionally concordant fractions (Figure S5D). Our modeling confirmed that the cross‐dataset overlap exceeds what could be expected by chance across seven out of the eight tested lineages. Highly significant overrepresentation profiles were observed within the glial and non‐neuronal infrastructure, prominently driven by astrocytes (351 shared genes; 79.2% same‐direction concordance; FDR = 2.09 × 10^−105^), oligodendrocyte precursor cells (85 shared genes; 83.5% concordance; 3.92 × 10^−30^), mature oligodendrocytes (150 shared genes; 77.3% concordance; FDR = 1.28 × 10^−11^), pericytes FDR = 3.07 × 10^−17^, and microglia (FDR = 2.01 × 10^−06^). Inhibitory neurons also revealed significant cross‐study overrepresentation (80 shared genes; 78.7% concordance; FDR = 1.90 × 10^−09^). Conversely, within the broader excitatory neuron population, the directional intersection slice did not surpass random biological baseline expectations (148 shared genes; 31.7% concordance; FDR = 0.99), capturing the high transcriptomic background and diverse baseline single‐cell projection states across distinct historical cohorts.

We also examined expression of genes manipulated in source studies within cell types identified in our integrated dataset. *Gls1*, knocked out in *CamKIIα* + neurons (Ji et al. 2023), was concordantly downregulated in excitatory neurons in our integrated analysis and in other dataset subsets (Chen et al. 2022; Pollina et al. 2023; Zhang et al. 2024) (Figure S6). *Npas4*, knocked out by Pollina et al. (2023), was downregulated in the corresponding excitatory neuron subset and in other dataset subsets (Ji et al. 2023; Chen et al. 2022; Zhang et al. 2024). This cross‐model and cross‐paradigm consistency supports the polygenic nature of ASD architecture, indicating that distinct genetic and environmental insults converge onto identical, cell‐type‐specific operational hubs.

### 3.4 Cell–Cell Communication Disturbances in ASD Mouse Models

Overall cell communication analysis revealed a highly interconnected network among the coordinated brain populations (Figure S7A). To demonstrate the presence of expected canonical patterns of cellular communication and provide a functional biological validation for our cluster annotations and integration architecture, we mapped several baseline homeostatic pathways across lineages (Figure S7B). First, the Vascular Endothelial Growth Factor (VEGF) network is heavily concentrated within non‐neuronal interfaces, primarily involving vascular cells and astro‐ependymal cells as the dominant sender‐receiver participants (Figure S7B1). Second, the Somatostatin signaling network acts as a highly restricted paracrine and autocrine axis, operating exclusively within inhibitory and excitatory neuronal subpopulations (Figure S7B2). Third, the Neuregulin (NRG) pathway displays a strict, unidirectional neuroglial crosstalk architecture, where signaling originates from neuronal populations and targets precursors and mature oligodendrocytes (Figure S7B3). Finally, the CX3C chemokine axis is uniquely restricted to a highly specific cellular link, connecting fractalkine‐expressing neurons to immune cells (microglia) (Figure S7B4). The preservation of these highly cell‐type‐specific, canonical neurobiological pathways confirms that the computational harmonization successfully retained the functional and spatial organizational properties of the mammalian brain tissue.

Comparing ASD to control conditions (second annotation level, Figure 3A) revealed a significant increase in interactions between inhibitory and excitatory neurons, suggesting general dysregulation in neuronal signaling potentially underlying ASD‐related hyperconnectivity. Detailed analysis at the third annotation level (neuronal subtypes only, Figure 3B) showed this increase was broad across most neuronal populations. Subtypes L4/5 IT and PALVB‐like neurons showed substantial increases in interaction strength in ASD (Figure 3C). Analysis of key signaling pathways with altered activity in ASD (Figure 3D) highlighted pathways that govern vital neurodevelopmental wiring processes: the Pleiotrophin (PTN) pathway regulates extracellular matrix (ECM) remodeling and neuroblast migration; the SLIT network, mediated by key ligand‐receptor interactions such as *Reelin* (Reln) and *Robo1*, orchestrates axonal guidance and synaptic adhesion dynamics; the Protein Tyrosine Phosphatase Receptor (PTPR) axis modulates neurite outgrowth and forms functional signaling complexes involved in cellular adhesion; and the Semaphorin 3 (SEMA3) cascade serves as a critical repulsive cue governing growth cone collapse and precise synaptic pruning, had increased interaction numbers.

**FIGURE 3:**
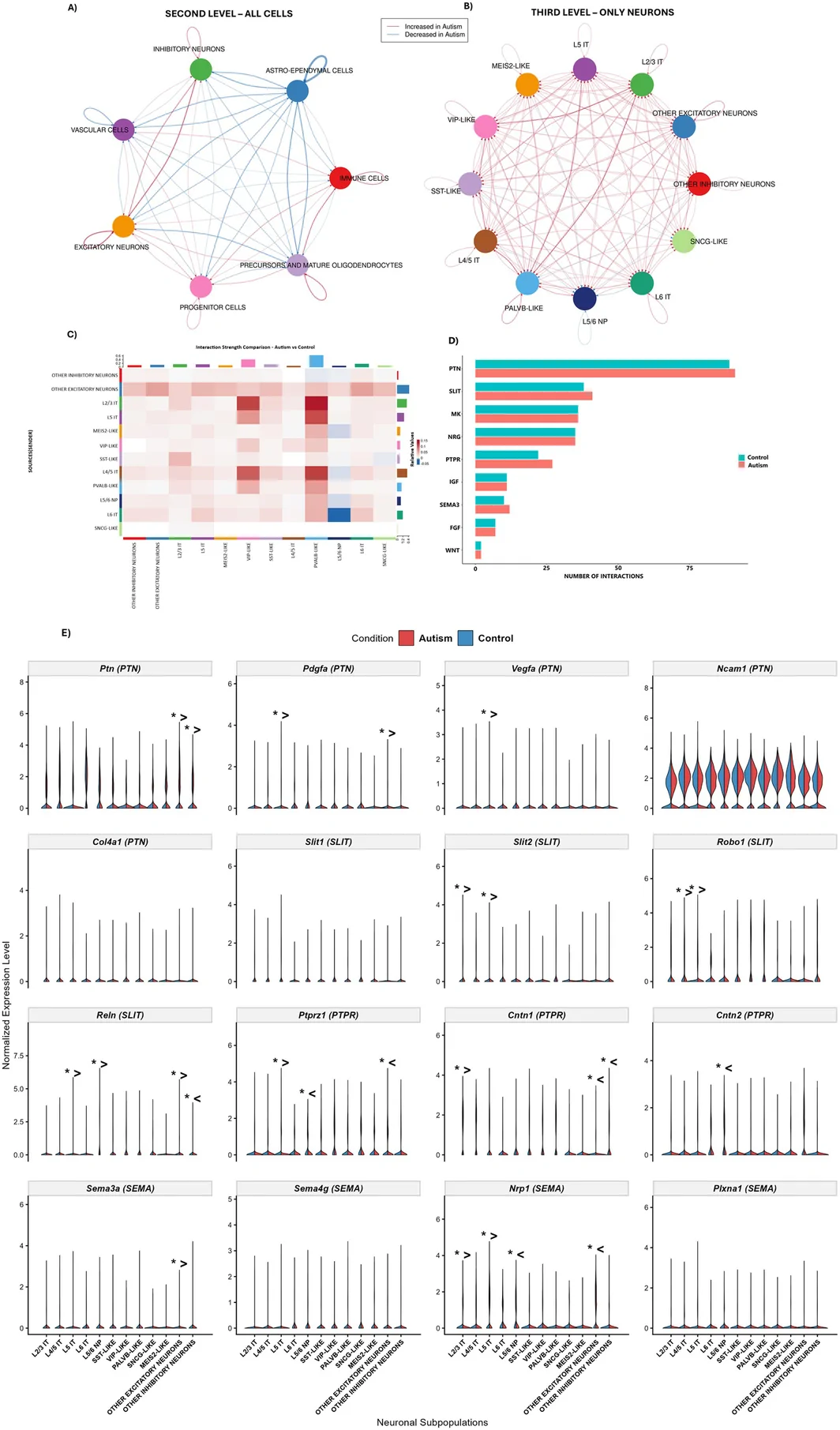
CellChat analysis of cellular communication in condition‐specific comparisons (ASD vs. Control). (A) Comparison of cellular communication networks between ASD and control samples at the second annotation level, including all cell types, shows increased communication in ASD (red edges) between inhibitory and excitatory neurons. (B) Interactions at the third annotation level, focusing only on neuronal subtypes, reveal that this increase in communication is consistent across most neuronal subtypes. (C) Interaction strength comparison heatmap illustrating the differences in signaling strength between ASD and control across all neuronal subtypes at the third annotation level. Red indicates stronger signaling in ASD, while blue represents stronger signaling in control. (D) Bar plot showing the number of interactions for key signaling pathways between ASD and control. Pathways such as PTN, SLIT, and NRG exhibit increased interactions in ASD. (E) Split violin plots showing the cell‐type‐specific single‐cell distribution magnitude (log2FC) for core genes belonging to dysregulated downstream pathways. Explicit statistical markers indicate target significance, with directional indicators (* > or * <) confirming whether a signaling component is significantly upregulated or downregulated in the Autism condition.

We cross‐referenced the core molecular components of the PTN, SLIT, PTPR, and SEMA cascades across fine‐grained neuronal subpopulations (Figure 3E). Within the PTN pathway, *Ptn* and *Pdgfa* displayed significant upregulation (*>) within post‐mitotic projection layers, including L4/5 IT and L6 IT neurons, accompanied by a localized induction of the downstream effector *Vegfa* in L4/5 IT streams. The SLIT network was characterized by a highly coordinated upregulation (* >) of *Slit2* and its canonical receptor *Robo1* within upper‐layer L2/3 IT and L4/5 IT projection neurons, while *Reln* expression shifted across distinct interneuron and projection subtypes. For the PTPR axis, the key receptor *Ptprz1* and its cell‐adhesion partners *Cntn1* and *Cntn2* revealed robust layer‐specific dynamics, marked by prominent induction (* >) in L4/5 IT layers contrasted by targeted downregulation (* <) in deeper cortical boundaries and interneuron sub‐lineages. Finally, the SEMA cascade demonstrated localized alterations, highlighted by the significant induction of *Sema3a* in other excitatory clusters alongside mixed, cell‐type‐specific regulation of its co‐receptor *Nrp1*.

Incoming signaling patterns (receiver signals) showed a global increase in pathway activity across neuronal subtypes in ASD, especially for PTN and SLIT (Figure S7C), with heightened receiver strength across most excitatory (e.g., L2/3 IT, L5 IT, L4/5 IT) and inhibitory (e.g., VIP‐LIKE, SST‐LIKE, PVALB‐LIKE) subtypes. PTPR and SEMA3 also showed increased reception, particularly in IT and L5/6 NP subtypes. Outgoing signaling patterns (sender signals) mirrored this, with ASD showing increased activity from key neuronal subtypes (Figure S7D). For PTN, PTPR, and SEMA3, enhanced sender activity appeared largely driven by excitatory clusters (L2/3 IT, L5 IT, L4/5 IT, L5/6 NP). SLIT showed a pattern with potentially increased contributions from various inhibitory subtypes in ASD. These findings suggest ASD is characterized by widespread alterations in neuronal communication, driven by increased activity in key signaling pathways and dysregulated sender‐receiver computational dynamics. Crucially, these predictive interactome signatures serve as hypothesis‐generating candidate pathways rather than direct proof of functional hyperconnectivity.

Given the key role of inhibitory and excitatory neurons in maintaining the balance of neuronal network activity, we concentrated on inhibitory neurons as senders and excitatory neurons as receivers, as shown in Figure 4. This choice was influenced by the observation that inhibitory neurons frequently play a role in shaping and modulating downstream signaling in neural circuits, and their dysregulation could lead to cascading effects on excitatory (Capogna et al. 2021), potentially contributing to the extensive communication changes seen in ASD. When we examined top ligand expression and distribution in inhibitory neurons (Figure 4A), *Pdgfa*, *Lsamp*, and *Adamts3* had high activity and broad expression, while *Wnt7a* and *Jam2* were more restricted. Ranking top ligands by AUPR and analyzing fold change (ASD vs. control, Figure 4B), *Pdgfa*, *Adamts3*, and *Lsamp* were most active, with significant regulation across multiple inhibitory neuron subtypes, despite low AUPR values (expected due to ASD's complex etiology). Ligands like *Jam2*, *Thy1*, and *Lpl* showed consistent downregulation across all inhibitory subtypes. Predicted target genes in excitatory neurons (Figure 4C) included *Pdgfa* targets *Arl4a* (upregulated in L5 IT and other excitatory neurons) and *Atad2* (downregulated in these groups) (Table S9). *Egr1*, a target of *Reln*, was significantly downregulated in L4/5 IT and L5 IT neurons. Ligand‐receptor interaction analysis (Figure 4D) prioritized ligands with high interaction potential. For *Bdnf*, its receptor *Ntrk2* showed no significant changes, but *Sort1* was significantly downregulated in L5 IT neurons (Table S9). Key receptors *Nrp1* and *Nrp2* were identified; *Nrp2* was consistently upregulated in L5 IT neurons, while Nrp1 showed a mixed regulation pattern across excitatory subtypes.

**FIGURE 4:**
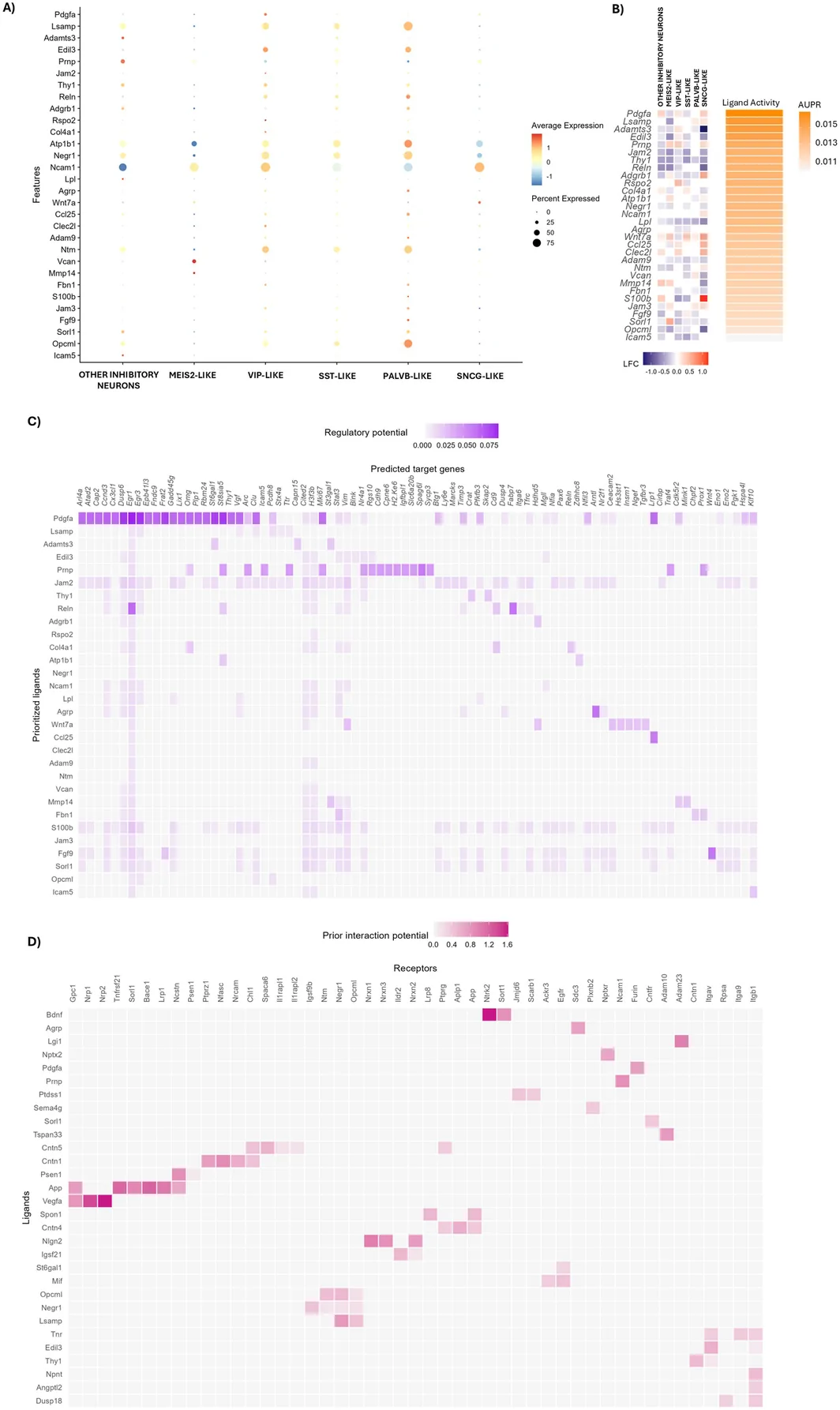
NicheNet analysis of ligand‐receptor interactions between inhibitory neurons (senders) and excitatory neurons (receivers). (A) Dot plot displaying the expression levels (average expression) and percentage of cells expressing the top ligands identified in inhibitory neurons, categorized by neuronal subtypes. Each dot's size indicates the proportion of expressing cells, while the color denotes the average expression level. (B) Heatmap ranking the top ligands based on the area under the precision‐recall curve (AUPR), their log fold change (LFC) in ASD compared to control, and their expression across inhibitory neuron subtypes. (C) Heatmap illustrates the predicted target genes in excitatory neurons (receivers) affected by the top ligands. The intensity of purple shading represents the regulatory potential of each ligand‐target gene interaction, reflecting the anticipated impact of ligand activity on target gene expression in the receiver cells. (D) Heatmap of the top receptors in excitatory neurons, displaying the prior interaction potential for each ligand‐receptor pair. The intensity of shading reflects the strength of the predicted interaction, emphasizing key ligand‐receptor interactions facilitating communication between inhibitory and excitatory neurons.

### 3.5 Convergence of DEGs of Integrated ASD Mouse Models and Clinically Relevant Genes

Lastly, we propose comparing our findings with existing literature detailing gene alterations associated with ASD in humans. First, we conducted an analysis comparing the DEGs identified in the third level of cell annotations with the SFARI database (Abrahams et al. 2013), which curates ASD‐linked genes scored 1 (strongest evidence) to 3 (emerging evidence) (Figure 5). Most matched genes were high‐confidence (Scores 1 or 2) (Figure 5A). Neuronal subtypes like L4/5 IT (16.5%), L2/3 IT (11.5%), L5/6 NP (10.8%), and Other Inhibitory Neurons (10.6%), along with Pericytes (10.1%), had a high percentage of SFARI matched genes among their DEGs. To formally evaluate whether the observed intersections between our differentially expressed profiles and ASD‐susceptibility loci were greater than expected by chance, we performed an overrepresentation analysis via a hypergeometric test across all lineages. The statistical test confirmed that our cell‐type‐specific molecular signatures are enriched with high‐confidence autism risk genes cataloged in the SFARI database. A statistical significance of 2.2 × 10^−16^ was achieved across multiple cortical and glial drivers, including Astrocytes (77 genes observed), Ependymal‐like cells (544 genes), L2/3 IT neurons (167 genes), L5 IT neurons (202 genes), and Mature Oligodendrocytes (296 genes). Furthermore, refined subpopulations also displayed substantial genetic overrepresentation, such as Oligodendrocyte Precursor Cells (*p* = 1.52 × 10^−12^) and Microglia (*p* = 1.01 × 10^−9^), mathematically demonstrating a robust genetic convergence, with the exception of SNCG‐LIKE with *p* = 0.059. These comprehensive overrepresentation outputs have been compiled into Table S17.

**FIGURE 5:**
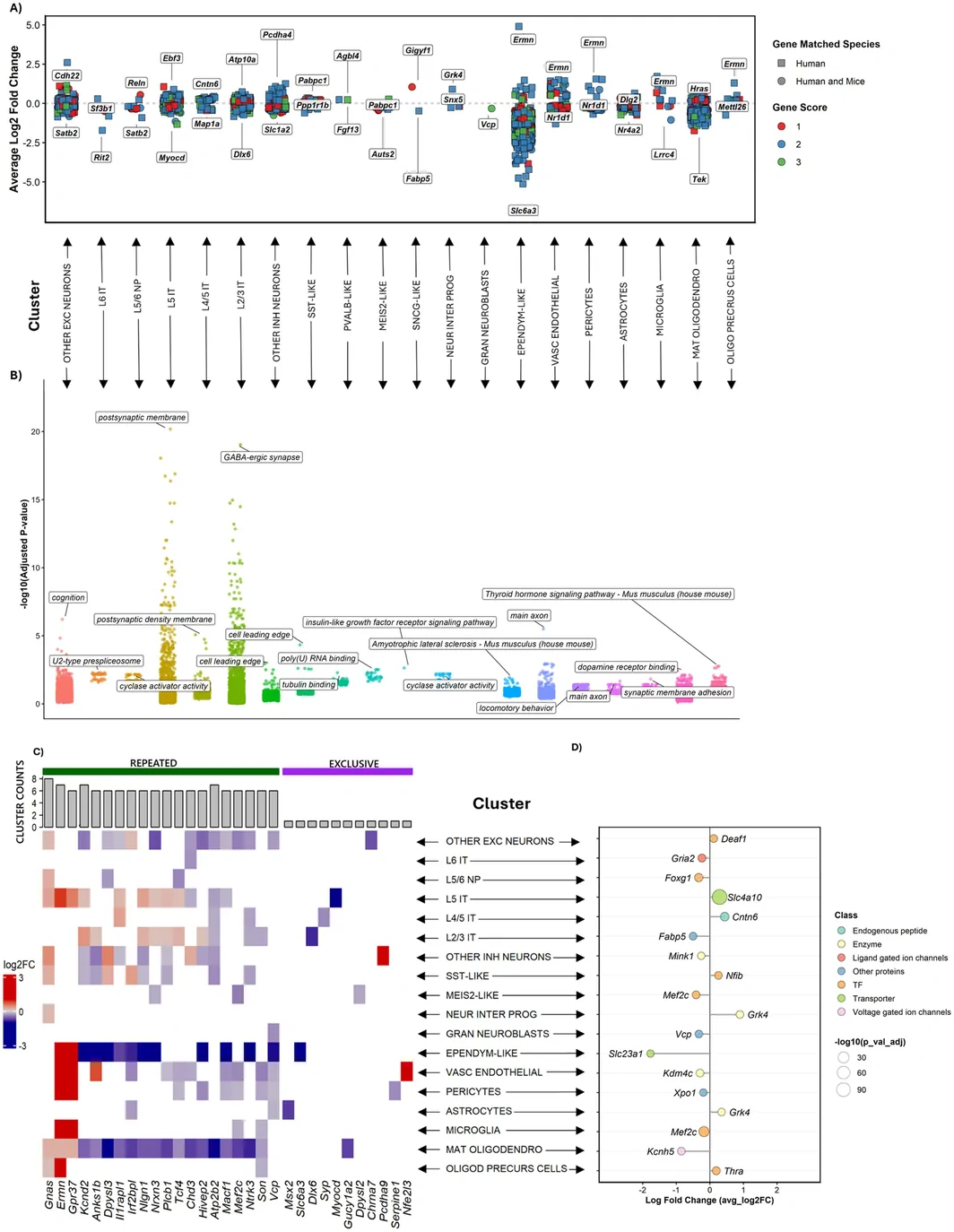
Matching DEGs with the SFARI database and functional analysis. (A) The scatter plot illustrates the average log2 fold change (avg_log2FC) of DEGs per cluster matched to the SFARI database. Geometric markers are substantially enlarged and optimized using high‐contrast solid filled designs. Points are color‐coded based on gene score (1–3), and shape‐coded by species evidence (bold square for human‐only clinical data, or circle for human and mouse intersection data) to guarantee cross‐species visual distinction. Genes with higher scores (1 or 2) indicate greater confidence as ASD‐associated genes. (B) Gene ontology analysis of matched DEGs displays the top enriched biological processes for each cluster. Points represent the significance (−log10 adjusted *p*‐value) of the GO terms, with certain significant terms related to ASD being highlighted. (C) The top 20 most shared and the top 10 unique DEGs across clusters by absolute log2FC, with bars representing the number of clusters in which each gene is either upregulated or downregulated in the heatmap, by avg_log2FC (−3, 3). (D) A lolliplot categorizes the top DEG (by avg_log2FC) for each cluster based on IUPHAR or TF classification. Categories include catalytic receptors, enzymes, transporters, transcription factors, and others, highlighting their functional roles.

*Ermn*, a known ASD‐associated gene, was upregulated in multiple non‐neuronal cell types (Table S18). GO/KEGG analysis of matched DEGs revealed significant ASD‐associated enrichment terms (Figure 5B, Table S19). In neuronal clusters, cognition (GO:0050890), learning/memory (GO:0007611), and regulation of membrane potential (GO:0042391) were enriched. In non‐neuronal clusters, antioxidant activity (GO:0016209; Microglia) and pathways of neurodegeneration (KEGG:mmu05022; Ependymal‐Like Cells, Mature Oligodendrocytes) were enriched. Many of those SFARI‐matched DEGs were shared across clusters (Figure 5C); *Kcnd2* and *Tcf4* showed complex, cell‐type‐specific regulation. Uniquely regulated genes included *Chrna7* (downregulated in other excitatory neurons), *Syp* (upregulated in L5‐IT), and *Pcdha9* (upregulated in other inhibitory neurons) (Figure 5C, left panel). Categorizing top DEGs by *p*_val_adjusted using IUPHAR and TF annotations (Figure 5D, right panel) highlighted *Gria2* (glutamate receptor subunit) downregulated in L6 IT excitatory neurons, and *Mef2c* (TF critical for neurodevelopment) downregulated in MEIS‐2 interneurons. Other significant ASD‐linked TFs like *Deaf1* (upregulated in Other Excitatory Neurons) and *Foxg1* (downregulated in L5‐6 NP) were also noted.

We next compared our integrated DEGs with single‐cell human post‐mortem data (Gandal et al. 2022) (Figure 6, Table S20). To provide statistical rigor to this cross‐species comparison and formally assess the significance of the directional concordance (same‐direction dysregulation, Up/Up or Down/Down), we applied a hypergeometric overrepresentation analysis against the human cortical datasets. The analysis revealed a massive, non‐random directional replication across major central lineages. In the prefrontal cortex (PFC), excitatory neurons shared 624 DEGs (320 consistently regulated; e.g., *CTXN3* up, *ADGRL4* down). Mature oligodendrocytes shared 607 DEGs (517 consistently regulated, significant correlation). Oligodendrocyte precursor cells also showed significant correlation (19 shared genes). In the parietal cortex, excitatory neurons had the highest overlap (1198 shared DEGs, 874 with significant consistent regulation; for example, *WNT5A* upregulated). Inhibitory neurons and mature oligodendrocytes had 59% and 79% of shared DEGs conserved, respectively, with significant correlations (Figure 6B). The occipital cortex showed the least consistent patterns (Figure 6C); excitatory neurons shared 931 DEGs but with weak correlation, and mature oligodendrocytes showed modest correlation, which was expected given the absence of occipital samples in all 11 ASD mouse datasets used in our integration. Aside from PFC Microglia, which remained unmixed as expected, virtually all critical neurobiological populations validated across species with statistical convergence, confirming that the recurrent signatures identified in our integrated object reflect genuine, biologically conserved paths of human autism pathology. These complete cross‐species statistical breakdowns are detailed in Table S21. We further investigated functional gene pathways related to shared DEGs modulated in the same direction between our integrated object and the parietal cortex in excitatory neurons, inhibitory neurons, and mature oligodendrocytes. SynGO analysis (Koopmans et al. 2019; Figure 6D,E; Table S22) for neurons revealed significant synaptic‐related BPs such as “translation at presynapse” and “translation at postsynapse” in both, with inhibitory neurons showing strong presynaptic enrichment in ribosomal protein genes (e.g., *RPL*, *RPS* genes). In excitatory neurons, presynaptic DEGs included *CACNA1A* and *UNC13A*; postsynaptic included *GRIN2B*, *CACNG2*, and *CAMK2A*. In inhibitory neurons, presynaptic DEGs included *ATP6V0A1* and *KCNMA1*; postsynaptic included *CAMK2A* and *NRG1*. For mature oligodendrocytes, Metascape analysis (Zhou et al. 2019) using DisGeNET (Figure 6F,G; Table S23) revealed enrichment of human disease‐associated genes like “Dyskinetic Syndrome,” “Neurodevelopmental Disorders,” “Epilepsy,” and “Mental Disorders”. A protein–protein interaction (PPI) network functional module (*DLGAP1, DLGAP2, NRXN1, NRXN3*) emerged, enriched in pathways like “Disruption of Postsynaptic Signaling by CNV” and “Neurexins and Neuroligins.”

**FIGURE 6:**
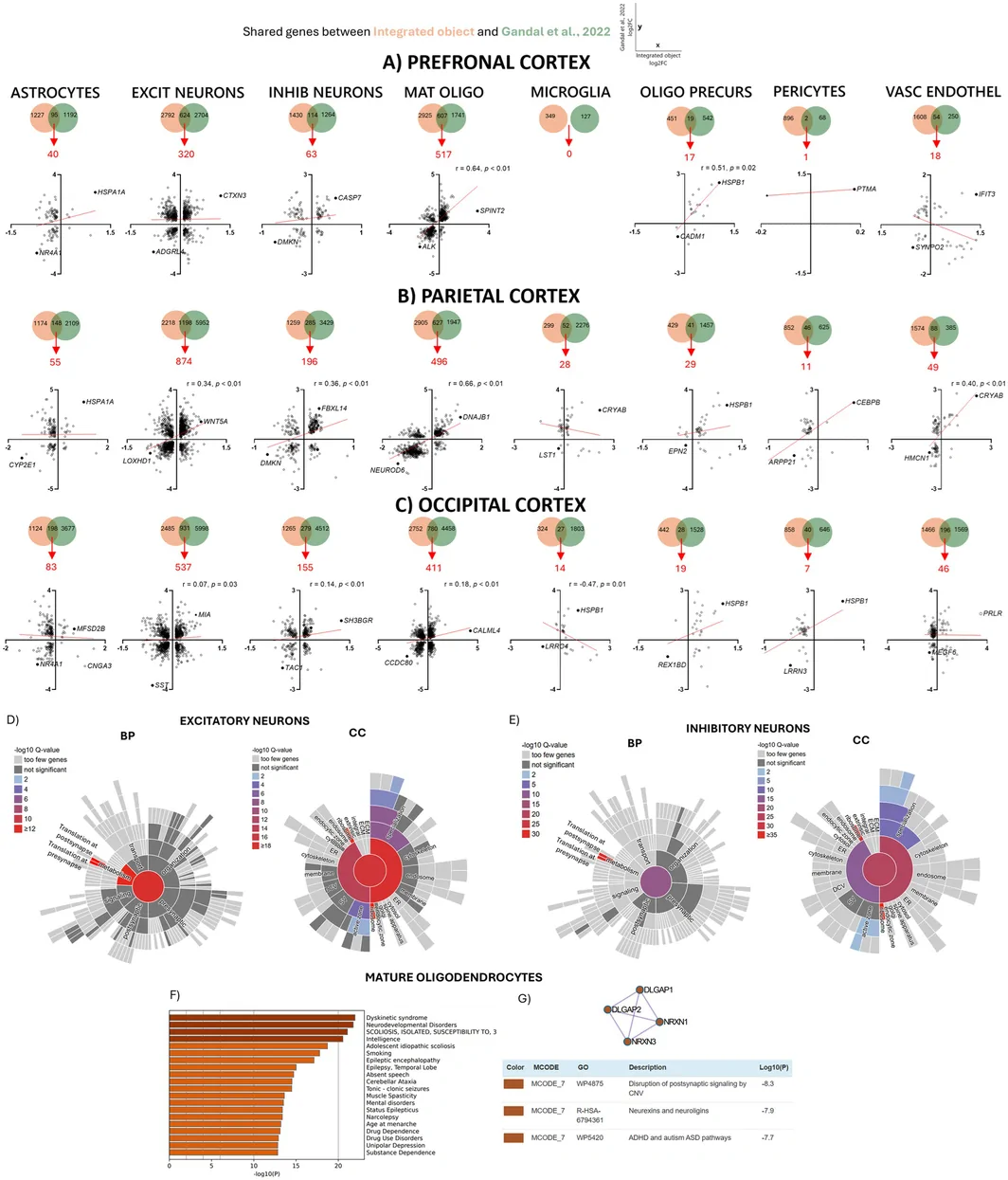
Identification and functional analysis of shared DEGs between the Integrated object and cortical transcriptomic data. (A–C) Venn diagrams illustrate the number of differentially expressed genes (DEGs) in common cell types between the integrated object and cortical transcriptomic data from Gandal et al. (2022). Intersections represent shared DEGs, with red numbers indicating genes regulated in the same direction. Asterisks denote significant correlations between datasets (*p* < 0.05). Corresponding correlation plots show the avg_log2FC for shared DEGs in the (A) Prefrontal cortex (PFC), (B) Parietal cortex, and (C) Occipital cortex. (D–G) Functional enrichment analysis of shared DEGs identified in the parietal cortex. (D, E) SynGO enrichment analysis of significant biological processes (BP) and cellular components (CC) for (D) excitatory and (E) inhibitory neurons. The color gradient indicates the significance level (−log10 Q‐value). (F) Metascape functional enrichment showing the top enriched human diseases for mature oligodendrocytes, with bars representing significance (−log10 *p*‐value). (G) Metascape network analysis for mature oligodendrocytes identifying key regulatory genes (DLGAP1, DLGAP2, NRXN1, and NRXN3) within enriched pathways.

## 4 Discussion

By systematically integrating scRNA‐seq datasets from multiple ASD models, we constructed a unified single‐cell reference for ASD‐related transcriptional dysregulation. While mechanisms such as E/I imbalance and glial dysfunction have been individually implicated in ASD, our work represents the first systematic integration demonstrating that these alterations are not model‐specific artifacts but robust, conserved signatures across diverse genetic and environmental etiologies. This approach allowed us to harmonize cell‐type annotations across studies and identify shared molecular signatures of ASD at a cellular level that transcend individual model idiosyncrasies. While this approach has previously been successfully utilized in the context of renal (Hrovatin et al. 2023) and metabolic disorders (Zhou et al. 2023), the present work represents, to our knowledge, the first effort to integrate scRNA‐seq data from neurodevelopmental disease mouse models.

When comparing ASD and control samples in our integrated dataset, we identified several DEGs across cell types at the third classification level. Notably, *Ttr*, which encodes transthyretin, was dysregulated in 13 clusters and emerged as the most upregulated gene in seven of them. *Ttr* encodes a non‐canonical thyroid hormone transporter primarily responsible for carrying thyroxine (T4) across the blood–brain barrier (Richardson et al. 2015). Thyroid hormones are essential for brain development, neuronal differentiation, and synaptogenesis (Schroeder and Privalsky 2014). Both maternal thyroid dysfunction during pregnancy (Kaplan et al. 2024) and individual thyroid dysfunction, particularly elevated T4 levels (Meng et al. 2024), have been linked to ASD. *Ttr* is also involved in retinol (vitamin A) transport through its interaction with retinol‐binding protein (Kang et al. 2018). Retinol is a crucial micronutrient for neurogenesis and neuronal plasticity (Shearer et al. 2012), and it has been implicated in ASD neurobiology (Liu et al. 2021), with emerging studies suggesting that vitamin A supplementation could serve as a potential therapeutic strategy for ASD (Guo et al. 2018).

Other consistently regulated genes include the murine pseudogene *BC023719*, a non‐coding RNA downregulated in eight cell types. Although non‐coding RNAs can regulate gene expression at both the transcriptional and post‐transcriptional levels, there is limited evidence regarding *BC023719* function, apart from its expression in the central nervous system (CNS) and retina (Blackshaw et al. 2004). In contrast, members of the *Olig* gene family, which encode basic helix–loop–helix (bHLH) TFs, also showed consistent regulation across clusters. Notably, *Olig2* and *Olig1* were upregulated in five non‐neuronal clusters, including oligodendrocyte precursor and mature cells. *Olig* genes play a critical role in oligodendrocyte development and neural lineage specification, and changes in *Olig2* expression have been linked to ASD (Szu et al. 2021). Studies in VPA‐induced ASD models have reported that *Olig2* expression in oligodendrocytes can be increased or decreased depending on age (Bronzuoli et al. 2018; Graciarena et al. 2019), while a network analysis of gene expression in cerebellar tissue of ASD patients also showed upregulation of *OLIG1* and *OLIG2* together with other oligodendrocyte markers (Zeidán‐Chuliá et al. 2016), further supporting the relevance of these TFs in oligodendrocytes' developmental dysfunction in ASD pathology.

Distinct transcriptional patterns emerged regarding DEG counts per cluster: pericytes, astrocytes, and ependymal cells showed predominant downregulation, while SST‐like interneurons and OPCs showed upregulation. Several genes positively enriched were also differentially regulated in ASD versus control comparisons in specific clusters, indicating cellular gain or loss of function. L4/5‐IT excitatory neurons, crucial for cortico‐cortical information relay (Im et al. 2022), showed a positive correlation between the gene enrichment and ASD regulation, suggesting intensified specific MF. For example, upregulation of synaptic adhesion molecule (*Cntn6, Nrxn1, Cdh12*) and scaffolding proteins (*Dlg2*) genes alter its synaptic stability and connectivity (Redies et al. 2012; Mercati et al. 2017; Cooper et al. 2024), while increased expression of genes coding ion channels (*Kcnip4, Hcn1*) might impact its firing properties, contributing to E/I imbalance (Marini et al. 2018; Ji et al. 2021). Conversely, astrocytes showed a negative correlation, with 98% of DEGs common with positively enriched genes found to be downregulated. Downregulation of *Aqp4* and *Kcnj10* likely impairs water/potassium homeostasis (Benga and Huber 2012; Morin et al. 2020; Bonosi et al. 2023); reduced *Gja1* compromises astrocyte network communication (Altas et al. 2024); *Glul* downregulation points to deficient glutamate‐glutamine cycling (Fan et al. 2023); and reduced *Atp1a2* suggests diminished ion transport for neurotransmitter uptake (Sugimoto et al. 2020). Such astrocyte dysfunction is increasingly recognized as a contributing factor in ASD pathophysiology (Cano et al. 2024), likely to create a less supportive environment for neurons, exacerbating neuronal network instability, and contributing to the overall E/I imbalance. While experimental validation of specific targets was beyond the scope of this integrative computational study, we reinforced our findings through pseudobulk validation (Figure S4). Moreover, in the original work by Donnard et al. (2022), they notably reported an astrocyte‐mediated exacerbation of excitatory‐inhibitory imbalance. Aligning with this, astrocytes showed the highest number of shared DEGs when comparing their dataset with our integrated results. This concordance extended to correlation analyses performed between data subsets (from our integration vs. Donnard et al. 2022 data subset), where neuronal and astrocyte populations exhibited the strongest correlations. This pronounced downregulation points toward significant deficits in astrocyte function within these models.

In mature oligodendrocytes, 871 DEGs were enriched, 91% being upregulated. Among these, *Pcsk1* (prohormone convertase 1/3), a key enzyme involved in endocrine and metabolic regulation (Stijnen et al. 2016) and linked to GI disorders comorbid with ASD was upregulated (Madra et al. 2021). *Ppia* (peptidylprolyl isomerase A), linked to inflammatory diseases and proposed as one distinguishing gene between ASD and controls (Hamzic et al. 2024), was also upregulated. *Jund*, involved in oxidative phosphorylation and upregulated in maternal immune activation models for ASD (Li et al. 2022), was also identified. This finding aligns with our GO enrichment analysis for mature oligodendrocyte DEGs, where “oxidative phosphorylation” emerged as the most significantly enriched BP. Oxidative phosphorylation is increasingly recognized as a critical pathway in ASD neurobiology, with evidence linking mitochondrial dysfunction to altered neuronal and glial energy metabolism in ASD (Carbonell et al. 2023).

Strong correlations between individual reference subsets and the integrated object for most cell types validated our approach, confirming convergent molecular alterations across diverse ASD models. Vascular cells were an exception, showing high significant correlation only with the VPA model (Zhang et al. 2024), suggesting model‐specific vascular changes. While research on the role of vascular health in brain development and ASD is still emerging (Ouellette et al. 2024), previous studies indicate that VPA affects vascular development during early life (Manzo et al. 2025) and has anti‐angiogenic effects in postnatal animals (Iizuka et al. 2018). Neuronal specific mutation models such as Glutaminase 1 deficiency in *CamkIIa* + cells (Ji et al. 2023) lacked significant vascular alterations, supporting this.

The E/I imbalance leading hypothesis in ASD (Uzunova et al. 2016) was supported by our cell–cell communication analysis showing increased neuronal communication, consistent with human single‐cell data (Zhao et al. 2023). NicheNet identified ligands and target genes aligning with pathways showing increased communication (PTN, SLIT, PTPR). *Pdgfa*, a top ligand, was upregulated, consistent with PTN pathway's role in growth factor signaling and ECM regulation (González‐Castillo et al. 2015). PTN can inhibit VEGFA signaling (Lamprou et al. 2020) (one predicted ligand), and blocking VEGFA can alleviate Fragile‐X syndrome cognitive impairments (Belagodu et al. 2017). Target genes *Ncam1* and *Col4a1* are also involved in ECM remodeling (Kuo et al. 2012; Vukojevic et al. 2020), further reinforcing the significance of these processes in neuronal dysregulation found in ASD. *Reln* (Reelin), another predicted ligand crucial for axon guidance and synapse organization (Faini et al. 2021), is also linked to the SLIT pathway (Gonda et al. 2020). *Reln* and *Robo1* (SLIT/ROBO pathway) modulate neuronal adhesion via N‐cadherin (Rhee et al. 2007; Matsunaga et al. 2017). *Adamts3*, a metalloproteinase able to inactivate Reelin (Ogino et al. 2017), has been previously linked to ASD through GWAS studies (Rexrode et al. 2024), reinforcing the idea that dysregulation of *Reln* processing may contribute to altered neuronal development in ASD (Scala et al. 2022).

Crucially, our analysis identified robust alterations in direct Semaphorin 3 components, characterized by the localized induction (*>) of the repulsive axon‐guidance ligand *Sema3a* alongside a highly coordinated, layer‐specific dysregulation of its core receptor *Nrp1* across intratelencephalic projection populations. This explicit involvement of canonical SEMA3 cascades complements the broader identification of related family members, such as *Sema4g*, further implicating semaphorin‐plexin repulsive cues and growth cone collapse pathways in the architecture of autism‐related circuit anomalies (Steele et al. 2022; Carulli et al. 2021). Although *Sema4g* function remains unclear, it may play a role in axon guidance (Belyk et al. 2015). Remarkably, a key receptor in the PTPR signaling pathway, *Ptprz1*, was found to be downregulated in L5/6 NP and other excitatory neurons, while its predicted ligands, *Cntn1* and *Cntn2*, were downregulated in other inhibitory neurons. Although *Ptprz1* and *Cntn1* are well known for their roles in neurite outgrowth and cell adhesion in glial cells (Mohebiany et al. 2013), their involvement in neuronal signaling remains largely unexplored. A recent study identified *Cntn2* as an interaction partner in a gene–gene *in silico* analysis for both *Cntn1* and *Ptprz1*, suggesting that these molecules form a functional signaling complex in patients with idiopathic generalized epilepsy (Lin et al. 2024). Given that epilepsy is a common comorbidity in ASD (Keller et al. 2017), this finding indicates that PTPR signaling dysregulation may contribute to both ASD and epilepsy‐related neural dysfunction.

Our findings of widespread alterations in ligand‐receptor pairs governing excitatory‐inhibitory communication align with previous transcriptomic meta‐analyses, which identified signatures of neural hyperexcitation and synaptic pseudo‐immaturity shared across multiple neuropsychiatric disorders (Murano et al. 2019). However, it is important to notice that transcriptomic inference captures steady‐state mRNA abundance rather than active protein translation, receptor phosphorylation, or synaptic vesicle release probability; these altered communication scores may instead capture alternative homeostatic or secondary scenarios. For instance, the heightened signaling density could reflect a compensatory homeostatic response enacted by the neural architecture to counteract pre‐existing circuit instability, or a secondary consequence of altered neuronal state and delayed transcriptomic maturation (Fan et al. 2024).

As anticipated, a significant overlap was found between our DEGs and SFARI database genes. *Ermn*, encoding an oligodendroglia‐specific protein, was upregulated across non‐neuronal populations; rare genetic variants leading to hypomethylation at the *ERMN* locus have been significantly associated with ASD patients (Homs et al. 2016), which corroborates with our findings of *Ermn* upregulation in non‐neuronal cells. *ATP2B2*, encoding calcium‐transporting ATPase‐2, an essential Ca^2+^ extrusion pump in neurons, was consistently downregulated across multiple neuronal subtypes. Variants in *ATP2B2* have been associated with ASD‐like phenotypes in transgenic mice, which present calcium extrusion dysfunction, motor impairments, and cerebellar atrophy (Poggio et al. 2023). Furthermore, *ATP2B2* loci polymorphisms have been consistently linked to ASD in large‐scale GWAS studies (Autism Spectrum Disorders Working Group of the Psychiatric Genomics Consortium 2017). Key TFs *Foxg1* and *Mef2c* were also identified. *FOXG1*, linked to severe developmental syndromes (Wong et al. 2019; Mazel et al. 2024), was downregulated in L5/6 NP neurons. *FOXG1* is crucial for cortical laminar organization, forming a complex with *Rp58* to repress genes like *Robo1, Slit3*, and *Reen* (found in our communication analysis), and haploinsufficiency of *Foxg1* in cortical neurons causes severe phenotypes associated with FOXG1 syndrome (Cargnin et al. 2018). *MEF2C*, associated with ASD and RTT‐like disorders, key for neurogenesis and synaptic pruning (Zhang and Zhao 2022), and was downregulated in MEIS2‐like interneurons; *MEIS2* mutations also link to RTT‐like features (Srivastava et al. 2018). Both *MEF2C* and *MEIS2* are directly implicated by chromosomal breakpoint mapping in patients with intellectual disability (Schluth‐Bolard et al. 2019). These findings highlight how TF‐mediated regulation may contribute to altered cortical organization and neurodevelopmental deficits observed in ASD pathophysiology, particularly in neuronal subtype‐specific contexts.

Our comparison with human postmortem data revealed conserved transcriptomic alterations, particularly in prefrontal and parietal cortices, which have been widely implicated in ASD (Wymbs et al. 2021; Leisman et al. 2023). The parietal cortex showed the highest consistency, with conserved regulation in excitatory/inhibitory neurons and mature oligodendrocytes. SynGO analysis showed enrichment of synaptic signaling and protein synthesis pathways (“translation at presynapse/postsynapse”) in neurons, highlighting potential vulnerabilities in synaptic function fundamental to neuronal communication. Altered presynaptic genes (such as *CACNA1A* in excitatory neurons) mutations of which are linked to neurodevelopmental disorders (Kramer et al. 2023), and *ATP6V0A1* in inhibitory neurons, vital for GABAergic function and implicated in epileptic encephalopathies (Bott et al. 2021) and postsynaptic genes (including the NMDA receptor subunit *GRIN2B*) an established ASD risk gene (Pan et al. 2015), and the AMPA receptor regulator *CACNG2* in excitatory neurons points toward disrupted signal reception and plasticity mechanisms (Lee et al. 2025); and the signaling molecule *NRG1* in inhibitory neurons, which regulates GABAergic circuit development (Navarro‐Gonzalez et al. 2021), along with consistent *CAMK2A* alterations (a key kinase in synaptic plasticity often affected in ASD; Yasuda et al. 2022), suggest disrupted neurotransmission, E/I balance, and plasticity. Enrichment in presynaptic active zones and postsynaptic density components further supports the hypothesis that abnormal synaptic architecture contributes to ASD pathophysiology. The strong finding of altered ribosomal protein gene expression (e.g., *RPL* and *RPS* genes), especially in inhibitory presynaptic datasets, aligns robustly with previous reports of widespread alterations in ribosomal gene expression and protein synthesis machinery in ASD patient tissues and cells (Lombardo 2021), suggesting that compromised synaptic protein homeostasis, affecting local translation crucial for synaptic maintenance and plasticity, may be a conserved molecular feature in ASD (Porokhovnik et al. 2015).

For mature oligodendrocytes, Metascape analysis using the DisGeNET platform demonstrated a significant enrichment of disease‐associated genes, particularly those connected to neurodevelopmental disorders, epilepsy, and mental disorders, which, considering their role in axonal myelination and neuronal connectivity, suggests that disruptions in their maturation and function could lead to deficits in neuronal circuit stability in ASD (Galvez‐Contreras et al. 2020). Moreover, the involvement of *NRXN* and *DLGAP* family genes in our analysis indicates that oligodendrocytes may directly influence synapse stabilization and plasticity, reinforcing previous findings linking white matter abnormalities in ASD to altered *NRXN* signaling in oligodendrocyte function (Proctor et al. 2015). These results further support the increasing recognition that glial dysfunction plays a crucial role in ASD beyond its traditional.

Despite the robustness of our integrative approach, several limitations must be acknowledged. First, the integration of scRNA‐seq and snRNA‐seq datasets, while necessary to maximize sample size, introduces potential biases due to differences in RNA composition between whole cells and nuclei. While QC thresholds were adjusted (1% mitochondrial for nuclei) and RPCA integration anchored shared biological states, subtle isoform‐level differences or cytoplasmic RNA depletion in nuclei may influence results, although the core cell‐type signatures remained robust (Luecken et al. 2022). Second, the disparity in developmental stages and brain regions across models introduces biological variability (Martinez‐Murcia et al. 2017). For instance, the high number of DEGs observed in ependymal cells (~7500 DEGs) is likely driven by study‐specific effects, as these cells were predominantly captured in specific datasets. However, we validated our integrated findings by comparing the integrated DEGs against those identified in individual datasets (Figure S5), observing a high degree of concordance across multiple cell types in specific high‐quality studies. Third, regarding the E/I imbalance, while our CellChat analysis indicates increased interaction strength, changes in the relative proportions of excitatory and inhibitory subtypes (as seen in some models) could also influence the total communication flux. Additionally, while our computational validation (Wilcoxon vs. Pseudobulk) confirms the statistical robustness of the DEGs, future studies employing spatial transcriptomics are warranted to validate the altered cell–cell communication patterns in situ and explore functional validation to elucidate further the causal relationships between the identified transcriptomic alterations and ASD pathogenesis.

Finally, comparisons with human postmortem data are inherently constrained by biological and technical variables that explain the more modest raw overlap counts or weaker correlations observed in specific cell types, such as microglia or cortical neurons. Transcriptomic alignment between species faces critical evolutionary and structural discrepancies, as human glia and neuronal subpopulations exhibit significantly higher morphological complexity, distinct gene expression profiles, and specialized functional responses compared to their murine counterparts. Furthermore, a substantial temporal and regional mismatch exists between the precisely controlled developmental windows captured in our mouse datasets and the highly variable chronological adult ages characteristic of human post‐mortem tissues. This developmental disparity fundamentally alters the baseline transcriptional landscape of highly plastic cell types like microglia and mature cortical layers. Beyond these biological variables, unavoidable technical discrepancies between datasets, including variations in post‐mortem intervals (PMI) within human samples, sequencing depth, and the differing capturing efficiencies of whole‐cell single‐cell versus single‐nucleus RNA‐seq platforms, inherently limit cross‐species correlation values.

In summary, our findings of increased interactions between inhibitory and excitatory neurons suggest widespread network‐level dysfunction, reinforcing the hypothesis of an altered excitatory‐inhibitory balance in ASD. Moreover, non‐neuronal cells like astrocytes and mature oligodendrocytes could play a major role in this dysfunction, as evidenced here and suggested by others. Comparative analyses with human postmortem datasets further validate these findings, demonstrating the translational relevance of our integrated approach. Only through this integrated approach is it possible to simultaneously increase the statistical power and relevance of our findings, as well as enable comparative analyses among different models. We believe that DEGs consistently regulated across different experimental paradigms in animal models, and which show similar patterns in corresponding cell types in ASD patient samples, are pivotal to cellular brain function and essential for understanding the neurobiology of ASD.

## Author Contributions

A.S.M. and J.V.N. conceived the project. J.V.N. and V.J.D. conducted data acquisition and performed the bioinformatic analyses. A.S.M. supervised the bioinformatic analysis. All authors analyzed and interpreted the data. The manuscript was written by J.V.N. and A.S.M., with input and edits from A.R.M. and V.J.D.

## Funding

This work was supported by FAPESP (Fundação de Amparo à Pesquisa do Estado de São Paulo) [Nos. 2019/27581‐0, 2021/14426‐6, 2024/15733‐8, 2024/14265‐0, and 2024/19422‐7], CNPq (Conselho Nacional de Desenvolvimento Científico e Tecnológico) [No. 315564/2023‐7] and Coordenação de Aperfeiçoamento de Pessoal de Nível Superior—Brasil (CAPES)—Finance Code 001.

## Conflicts of Interest

A.R.M. is a co‐founder and has an equity interest in TISMOO, a company dedicated to genetic analysis and human brain organogenesis, focusing on therapeutic applications customized for autism spectrum disorders and other neurological disorders' origin genetics. The terms of this arrangement have been reviewed and approved by the University of California, San Diego, following its conflict‐of‐interest policies.

## Supporting information


**Figure S1:** Quality control metrics of the integrated single‐cell dataset. Violin plots displaying the distribution of key quality control metrics: number of detected genes (nFeature_RNA), total unique molecular identifier (UMI) counts (nCount_RNA), and percentage of mitochondrial gene expression (percent.mt). (A) Distribution of QC metrics stratified by the original reference study (Reference), highlighting dataset‐specific variability handled during integration. (B) Detailed distribution of QC metrics across each individual sample (orig.ident). Cells were filtered based on adaptive thresholds (5 MADs) for library size and complexity, and a fixed threshold for mitochondrial content (< 10% for single‐cell data, < 1% for single‐nucleus data) to exclude low‐quality cells and debris prior to downstream analysis.

**Figure S2:** Hierarchical dendrogram illustrating the clusterization process up to the fourth level. Levels 1, 2, 3, and 4 are shown, with the second and third levels annotated with the most differentially expressed gene for each cluster, while in the fourth level we maintained a functional nomenclature or added the most differentially expressed gene in that cluster.

**Figure S3:** Quantitative Assessment of Dataset Integration Quality and Spatiotemporal Biological Preservation. (A) Neuroanatomical brain area distribution: Stacked horizontal bar plot illustrating the percentage representation of distinct anatomical regions (Cortex, Hippocampus, Prefrontal Cortex, Striatum, and Telencephalon Medial Pallium) within each integrated Level 3 cluster. (B) Developmental stage representation: Stacked horizontal bar plot depicting the percentage distribution of chronological developmental timeframes (Embryo, Early Postnatal, Adolescence, and Adult) contributing to each annotated cell identity, demonstrating the structural preservation of temporal lineages post‐integration. (C) High‐resolution dataset mixing (iLISI)—Level 3 Subtypes: Distribution of integration local inverse Simpson's index (iLISI) scores calculated across all refined cellular annotations. Teal and blue horizontal bars represent the mean iLISI score $\pm$ standard deviation (SD), orange dots denote median values, and the vertical red dashed line indicates the baseline threshold for an unmixed state (1.0). High scores reflect robust batch homogenization and dataset mixing within local neighborhoods.

**Figure S4:** Methodological validation of differential expression analysis. (A) Single‐Cell vs. Pseudobulk Expression Profile Correlations: Correlation scatter plots comparing the Log2 Fold Changes (Log2FC) obtained from single‐cell level analysis (Wilcoxon rank‐sum test, x‐axis) versus sample‐level pseudobulk aggregation analysis (DESeq2, y‐axis) across representative cellular subsets. The red dashed line represents the linear regression fit. Pearson correlation coefficients (R) and *p*‐values are indicated for each cluster. (B) Magnitude and distribution of DisGeNet overlapping signatures: Jitter plot tracking the distribution of pseudobulk Log2 Fold Changes for core disease‐linked genes identified through enrichment mapping across Level 3 cell‐type clusters. Individual dots represent distinct intersecting genes, color‐coded along a gradient according to their exact sample‐level ‐log10 *p*‐value significance profile.

**Figure S5:** Comparative analysis of differentially expressed genes across integrated object and individual datasets in the single‐cell ASD Study. (A) Correlation analysis of DEGs between the integrated dataset and subsets from individual reference studies included in the integration. Each dot represents a cellular cluster, with dot size indicating statistical significance (−log10(*p*‐value)) and color intensity reflecting the strength of the correlation (scale: −0.3 to 0.9). (B) Normalized Pairwise Directional Concordance Matrix: dot plot matrices demonstrating pairwise transcriptomic conservation across all seven major cellular classes evaluated in this study, ordered programmatically by dataset representation strength. To eliminate total DEG count biases, the dot size represents the exact percentage of normalized directional concordance (the ratio of co‐upregulated or co‐downregulated targets divided by the total shared overlap between specific dataset pairs). The color intensity maps the absolute count of directionally consistent genes using a continuous log10 scale. (C) Heatmap showing the log2FC of modulated genes in each genetic paradigm model for ASD in the subset of the pharmacological model of valproic acid (VPA) subset in our integrated object. (D) Venn diagrams compare DEGs from the integrated dataset with those found in the selected original reference study (Donnard et al. 2022). DEGs from the Donnard et al., dataset were filtered for both statistical significance (adj_*p* value < 0.05) and an average log2 fold change > 0.25, ensuring robust comparisons. To formally demonstrate signal conservation beyond random chance, each cluster features its corresponding Benjamini‐Hochberg False Discovery Rate (FDR) derived from a formal Hypergeometric Enrichment Test evaluating directionally concordant components against a standard conservative background gene universe. Each diagram illustrates the overlap of DEGs for specific cellular clusters and red numbers indicating the % of shared DEGs that are regulated in the same direction

**Figure S6:** Heatmaps depicting differentially expressed genes in the integrated object and the subset of each study across distinct cell types: Each column represents a different reference (study), while each row corresponds to a gene of interest (modulated in each model). The color scale reflects the avg_log2FC values, ranging from blue (negative) to red (positive), with white cells indicating non‐significant or absent data for that gene in each reference. Each highlighted square indicates the correspondence of the modulated gene to the reference in that column.

**Figure S7:** CellChat Analysis of Cellular Communication in the Integrated Dataset and Condition‐Specific Comparisons (Autism vs. Control). (A) Overall cellular communication network for all cell types in the integrated dataset, independent of autism or control conditions. Nodes represent cell types, and edge thickness reflects the strength of signaling interactions. (B) Validation sub‐networks of canonical signaling channels: Heatmap matrices mapping communication probabilities (probabilities driven by the law of mass action) for specific homeostatic pathways to validate cell annotation fidelity: (B1) VEGF signaling network: Isolation of vascular and astro‐ependymal cells as the primary participants governing endothelial and barrier signaling. (B2) SOMATOSTATIN signaling network: Visualization of a pathway highly restricted to mature inhibitory and excitatory neuronal interactions. (B3) NRG signaling network: Unidirectional signaling axis moving from neuronal classes toward precursors and mature oligodendrocytes. (B4) CX3C signaling network: Specialized, highly specific neuroimmune link mapping signaling exclusively from neurons to immune cells (microglia). (C) Comparative outgoing signaling patterns: Heatmap panel detailing sender cell profiles across fine‐grained Level 3 neuronal subtypes in Control (left) versus Autism (right) conditions, tracking relative signaling strength changes across all active signaling pathways. (D) Comparative incoming signaling patterns: Heatmap panel detailing receiver cell responses across Level 3 neuronal subtypes in Control (left) versus Autism (right) conditions, identifying target cell‐type sensitivity and altered pathway recruitment in the autism phenotype.

**Table S1:** Summary and distribution of putative doublets identified and removed across individual samples and cell types using scDblFinder.

**Table S2:** Quantitative assessment of dataset integration quality reporting subpopulation‐specific integration Local Inverse Simpsons Index (iLISI) scores across all Level 3 cell types.

**Table S3:** Hierarchical cell‐type classification scheme across all five successive clustering levels (first to fifth level), defining lineage and subtype annotations from major cellular classes down to refined subclusters.

**Table S4:** Proportional distribution of cells across developmental stages (embryo, early postnatal, adolescence, and adult) and brain regions for each cell‐type cluster at the third level of hierarchical annotation.

**Table S5:** Cell counts and percentage distribution contributed by each independent reference study across hierarchical classification levels (first and second levels).

**Table S6:** Canonical gene markers and cell‐type‐specific expression profiles utilized for hierarchical cell identification at Level 3.

**Table S7:** Positive marker genes and enriched transcriptional profiles identified per cell cluster ranked by average log2 fold change.

**Table S8:** Gene Ontology (GO) and KEGG pathway enrichment analysis summary for cluster‐enriched marker genes across all cell types.

**Table S9:** Differentially expressed genes (DEGs) identified between ASD and control conditions across Level 3 cell types calculated via Wilcoxon rank‐sum test.

**Table S10:** Overlap and Spearmans correlation metrics between differentially expressed genes and cell‐type‐enriched marker genes across clusters.

**Table S11:** Functional classification of differentially expressed genes based on IUPHAR/BPS pharmacological families and transcription factors.

**Table S12:** Gene Ontology (GO) and KEGG pathway enrichment analysis of differentially expressed genes between ASD and control conditions per cell type.

**Table S13:** Sample‐level pseudobulk differential expression analysis using DESeq2, effect‐size concordance correlations, and overlap with single‐cell Wilcoxon DEGs.

**Table S14:** Hypergeometric enrichment test and disease‐association analysis of pseudobulk DEGs against the DisGeNET database across cell clusters.

**Table S15:** Pseudobulk‐specific Gene Ontology enrichment analysis detailing overlapping biological processes in oligodendrocyte and neuronal lineages.

**Table S16:** Pairwise correlation and shared DEG concordance metrics between the integrated dataset and individual reference cohorts at Level 2 classification.

**Table S17:** Hypergeometric overrepresentation analysis evaluating the statistical enrichment of cell‐type‐specific DEGs with high‐confidence SFARI autism risk genes.

**Table S18:** Comprehensive list and expression profiles of SFARI‐matched differentially expressed genes across all cellular lineages.

**Table S19:** Functional Gene Ontology (GO) and KEGG pathway enrichment analysis specifically performed on SFARI‐matched DEGs per cluster.

**Table S20:** Intersection, directional concordance rates, and effect‐size correlation metrics between integrated mouse DEGs and human post‐mortem cortical single‐nucleus RNA‐seq data.

**Table S21:** Statistical breakdown of cross‐species hypergeometric overrepresentation analyses across prefrontal, parietal, and occipital cortical regions.

**Table S22:** SynGO synaptic functional enrichment analysis of directionally concordant shared DEGs in excitatory and inhibitory neurons.

**Table S23:** Metascape functional enrichment and protein–protein interaction (PPI) network module analysis for shared human‐mouse DEGs in mature oligodendrocytes.

**Table S24:** Accession numbers, repositories, and detailed metadata for all publicly available single‐cell and single‐nucleus RNA‐sequencing datasets analyzed in this study.

## Data Availability

All single‐cell RNA‐sequencing datasets analyzed in this study are publicly available; accession numbers and repository details are listed in Table S24. The processed Seurat object and the R scripts used for integration, quality control, and differential expression analysis are available on Zenodo [10.5281/zenodo.18248303] upon request. Images provided by Servier Medical Art (https://smart.servier.com/), licensed under CC BY 4.0 (https://creativecommons.org/licenses/by/4.0/).
